# Regulatory RNAs and the HptB/RetS signalling pathways fine-tune *Pseudomonas aeruginosa* pathogenesis

**DOI:** 10.1111/j.1365-2958.2010.07146.x

**Published:** 2010-04-12

**Authors:** Christophe Bordi, Marie-Cécile Lamy, Isabelle Ventre, Elise Termine, Abderrahman Hachani, Sandy Fillet, Béatrice Roche, Sophie Bleves, Vincent Méjean, Andrée Lazdunski, Alain Filloux

**Affiliations:** 1 Laboratoire d'Ingénierie des Systèmes Macromoléculaires, UPR9027, CNRS-IMM, Université de la Méditerranée31 Chemin Joseph Aiguier, 13402 Marseille cedex 20, France; 2 Imperial College London, Division of Cell and Molecular Biology, Centre for Molecular Microbiology and Infection, South Kensington CampusFlowers Building, London SW7 2AZ, UK; 3 Laboratoire de Chimie Bactérienne, UPR9043, CNRS-IMM, Université de la Méditerranée, 31 Chemin Joseph Aiguier13402 Marseille cedex 20, France

## Abstract

Bacterial pathogenesis often depends on regulatory networks, two-component systems and small RNAs (sRNAs). In *Pseudomonas aeruginosa*, the RetS sensor pathway downregulates expression of two sRNAs, *rsmY* and *rsmZ*. Consequently, biofilm and the Type Six Secretion System (T6SS) are repressed, whereas the Type III Secretion System (T3SS) is activated. We show that the HptB signalling pathway controls biofilm and T3SS, and fine-tunes *P. aeruginosa* pathogenesis. We demonstrate that RetS and HptB intersect at the GacA response regulator, which directly controls sRNAs production. Importantly, RetS controls both sRNAs, whereas HptB exclusively regulates *rsmY* expression. We reveal that HptB signalling is a complex regulatory cascade. This cascade involves a response regulator, with an output domain belonging to the phosphatase 2C family, and likely an anti-anti-σ factor. This reveals that the initial input in the Gac system comes from several signalling pathways, and the final output is adjusted by a differential control on *rsmY* and *rsmZ*. This is exemplified by the RetS-dependent but HptB-independent control on T6SS. We also demonstrate a redundant action of the two sRNAs on *T3SS* gene expression, while the impact on *pel* gene expression is additive. These features underpin a novel mechanism in the fine-tuned regulation of gene expression.

## Introduction

In the course of infection, bacterial pathogens are subjected to changing conditions and stress to which they should respond by inducing or repressing virulence genes. Bacteria have evolved sensory systems, including two-component regulatory systems (TCSs). These systems involve a histidine kinase sensor protein, which detects environmental stimuli. Perception of an environmental cue by the sensor results in autophosphorylation and transfer of the phosphoryl group onto a cognate response regulator (RR), which most frequently binds DNA to control gene expression. In many cases, the activation of the RR by the sensor may transit through a Histidine phosphotransfer (Hpt) protein, which is acting as a phosphorylation relay. This is the case with LuxU, a *Vibrio cholerae* Hpt, which is targeted by three different kinases, CqsS, LuxN and LuxQ (Tu and Bassler, 2007). After transiting through LuxU, the phosphate is transferred onto a single RR, LuxO.

*Pseudomonas aeruginosa* is a Gram-negative bacterium that is responsible for numerous nosocomial infections. Genome mining revealed about 120 genes encoding histidine kinase sensors or RRs (Rodrigue *et al*., 2000). Moreover, only three genes encoding Hpt modules, namely *hptA*, *hptB* and *hptC*, were identified. In *P. aeruginosa*, some TCS pathways are involved in virulence or biofilm formation. The hybrid sensors RetS and LadS have been shown to be involved in the transition between chronic and acute infections by antagonistically controlling expression of genes involved in virulence, such as the type III secretion system (T3SS), or genes that are required for biofilm formation, such as those involved in polysaccharide synthesis (Goodman *et al*., 2004; Laskowski *et al*., 2004; Ventre *et al*., 2006). The two sensors appeared to intersect with another TCS formed by the GacS/GacA pair, in which GacS is an unorthodox sensor and GacA an RR. The GacS/GacA system was shown to be important for *P. aeruginosa* virulence and under defined conditions to regulate expression of the quorum sensing signal homoserine lactone C4-HSL (Reimmann *et al*., 1997; Rahme *et al*., 2000).

Recent progress in the understanding of regulatory mechanisms revealed that a quick and tight mode of regulation to modulate gene expression may involve small RNAs (sRNAs) (Romby *et al*., 2006; Toledo-Arana *et al*., 2007; Valverde and Haas, 2008). It was shown that RRs influence expression of sRNAs, which in turn promote or inhibit the translation of target mRNAs. Most sRNAs act using an antisense mechanism; however, some other sRNAs, such as CsrB and CsrC, display multiple GGA motifs, which are targets for a translational repressor, CsrA, in *Escherichia coli* and *V. cholerae* (Weilbacher *et al*., 2003; Lenz *et al*., 2005). The out-titration of CsrA by the sRNAs results in the expression of genes that are otherwise negatively controlled by this translational repressor and are required for virulence, biofilm formation and host interaction.

Two *P. aeruginosa* sRNAs are extremely well described, namely RsmY and RsmZ. These sRNAs act by titrating the RNA binding protein RsmA, which is a close homologue of the *E. coli* and *V. cholerae* CsrA. Just like CsrA, RsmA specifically binds to GGA motifs located in target mRNAs. RsmA negatively controls the expression of quorum sensing and several virulence factors (Pessi *et al*., 2001; Heurlier *et al*., 2004; Burrowes *et al*., 2006; Kay *et al*., 2006; Brencic and Lory, 2009). In particular, it was found to bind directly on transcripts encoding hydrogen cyanide synthesis components (Pessi *et al*., 2001) and more recently on transcripts encoding type VI secretion system (T6SS) components (Brencic and Lory, 2009). Importantly, in *P. aeruginosa* the production of RsmY and RsmZ is controlled by GacA (Kay *et al*., 2006; Brencic and Lory, 2009). We have previously shown that expression of *rsmZ* is positively controlled by the LadS pathway and negatively by the RetS pathway (Ventre *et al*., 2006). Overall, upregulation of *rsmZ* appears to promote bacterial biofilm formation and to prevent cytotoxicity.

LadS and RetS are hybrid sensors, and may require an Hpt module to transfer their phosphate onto a cognate RR. However, it was recently shown that RetS acts in a fairly unusual manner, by forming heterodimers with GacS and preventing the activation of the GacS/GacA pathway (Goodman *et al*., 2009). In this study, we observed that an *hptB* mutant displayed very similar phenotypes to a *retS* mutant. However, we present detailed evidence showing that despite these similarities, the HptB and RetS pathways are distinct. Although both pathways terminate on the GacA RR, HptB signalling controls expression of *rsmY* only, whereas RetS signalling modulates both *rsmY* and *rsmZ* gene expression. This subtle difference results in a significant difference in the control of target genes in the Gac/Rsm pathway.

## Results

### The hyperbiofilm phenotype of an *hptB* mutant is linked with the expression of *pel* genes

**Fig. 1:**
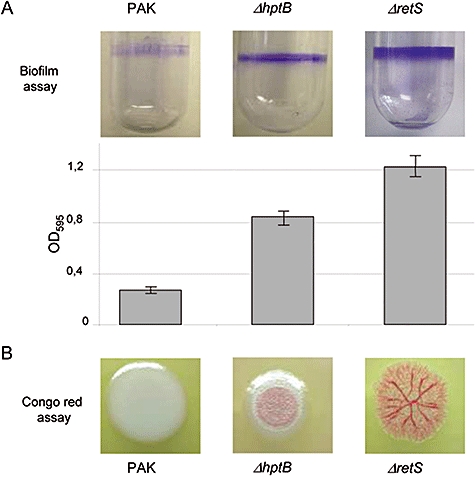
Comparison between PAKΔ*hptB* and PAKΔ*retS* mutants for biofilm formation and exopolysaccharide production. A. Glass tube assay showing biofilm formation (upper part). Quantification of the crystal violet-stained adherence ring formed in the glass tube (lower part). Each experiment was repeated three times. The error bars indicate standard deviations. The name of the tested strain is indicated above each panel. B. Bacterial colony staining on Congo red-containing agar plates. The name of strains used is indicated under each panel.

**Fig. 2:**
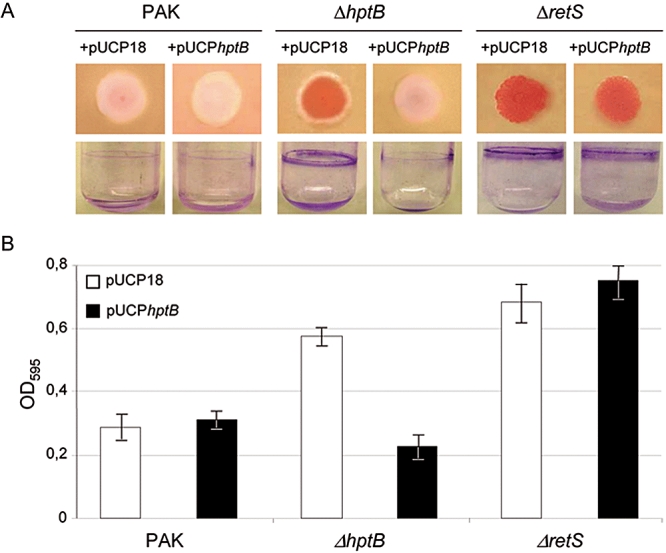
Influence of *hptB* overexpression in PAK, PAKΔ*hptB* or PAKΔ*retS* strains, on biofilm formation and exopolysaccharide production. A. Bacterial colony staining on Congo red-containing agar plates (upper row) and glass tube assay showing biofilm formation (lower row). The name of the tested strains is indicated above each panel. B. Quantification of the adherence ring formed in the glass tube. Each experiment was repeated three times. The error bars indicate standard deviations. The name of the strains used is indicated under each bar. Filled bars correspond to strains carrying pUCP*hptB* whereas open bars correspond to strains carrying pUCP18. The pUCP*hptB* allowed overexpression of the *hptB* gene cloned into the pUCP18 vector.

Preliminary studies by Hsu and colleagues suggested that an *hptB* mutant synthesizes and disintegrates biofilm at a higher rate as compared with the PAO1 wild-type strain (Lin *et al*., 2006; Hsu *et al*., 2008). Here, we engineered a deletion of the *hptB* gene (PA3345 at http://www.pseudomonas.com) in the *P. aeruginosa* PAK strain, yielding PAKΔ*hptB* (*Experimental procedures*; Table S1). The biofilm phenotype was tested in microtitre dishes or glass tubes as previously described (Ventre *et al*., 2006). The *hptB* mutant has a hyperbiofilm phenotype compared with PAK (Fig. 1A), which was very similar to one previously reported for the PAKΔ*retS* mutant (Goodman *et al*., 2004) (Fig. 1A). The hyperbiofilm phenotype in the *retS* mutant is linked to overproduction of exopolysaccharides, therefore we determined whether this was also the case with the *hptB* mutant. The *retS* and *hptB* mutants grown on plates containing Congo-Red dye displayed a strong staining, thus revealing polysaccharide production (Fig. 1B). The staining was stronger with the *retS* mutant when compared with the *hptB* mutant. Introduction of the *hptB* gene cloned in the pUCP18 plasmid (pUCP*hptB*), into the *hptB* mutant (PAKΔ*hptB*), resulted in restoration of biofilm and polysaccharide production (Fig. 2), confirming that the phenotype is due to the *hptB* deletion.

**Fig. 3:**
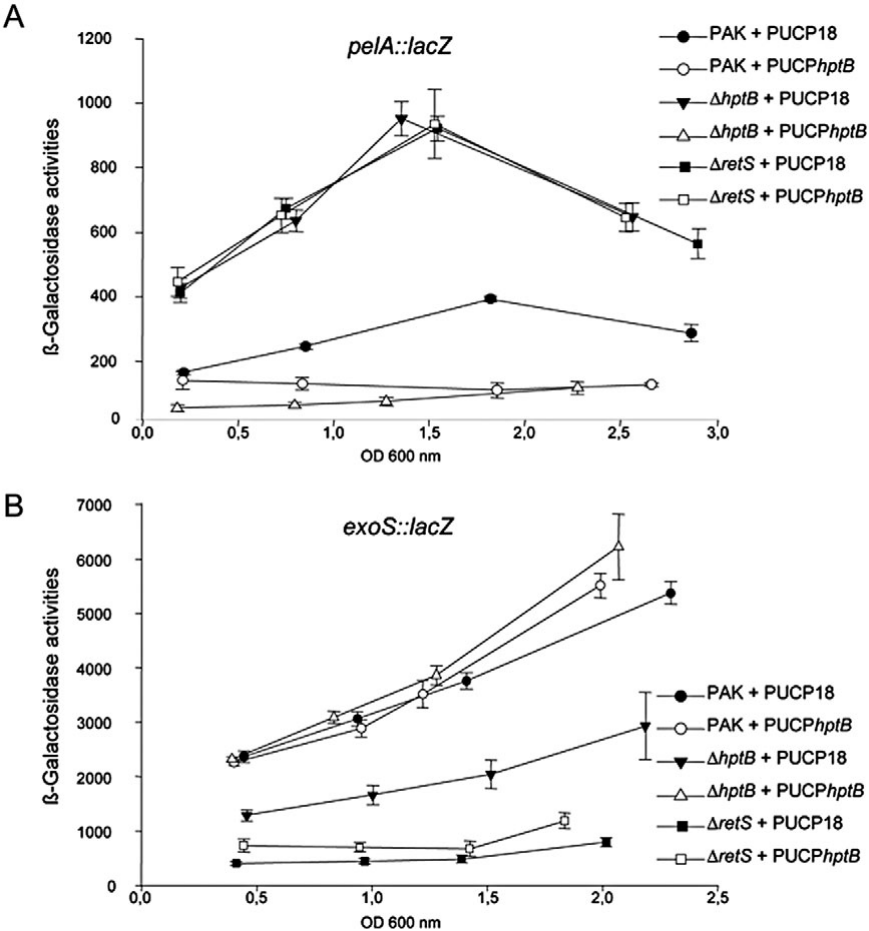
Expression of *lacZ* transcriptional fusion in PAK, PAKΔ*hptB* or PAKΔ*retS* strains. Activity was recorded at different growth stages. A. Activity of the *pelA–lacZ* transcriptional fusion. B. Activity of the *exoS–lacZ* transcriptional fusion (carried on pBS307). Open signs correspond to strains carrying pUCP*hptB* whereas filled signs correspond to strain carrying pUCP18. β-Galactosidase activities are expressed in Miller units. Values are averages of at least three independent experiments.

Congo red staining has previously been reported as being linked to overexpression of the *pel* genes (Friedman and Kolter, 2004; Vasseur *et al*., 2005). We introduced a *pelA–lacZ* transcriptional fusion carried on pMP220 into the PAK and PAKΔ*hptB* strains (Ventre *et al*., 2006). The strains were grown in Luria broth (LB) at 37°C. The level of β-galactosidase activity measured at different growth stages revealed a higher activity of the *pelA* promoter in the *hptB* mutant in comparison to the parental PAK strain (Fig. 3A). The maximal induction of the *pelA* promoter was reached at an OD_600_ of 1.4. Expression level was increased by 2.9-fold in the *hptB* mutant as compared with PAK (Fig. 3A). Upon introduction of a plasmid carrying the *hptB* gene (pUCP*hptB*), expression of the *pelA* transcriptional fusion was strongly inhibited both in the parental and the *hptB* mutant (Fig. 3A). These observations clearly suggest that HptB signalling negatively controls *pel* gene expression. Finally, the causal link between the biofilm phenotype of the *hptB* mutant and the increased level in *pel* gene expression was established. Indeed, introduction of a *pelB* mutation in the *hptB* mutant abolished the hyperbiofilm phenotype and congo-red staining (Fig. S1; *Experimental procedures*; Table S1).

### Overlap between the *hptB* and *retS* mutant transcriptomes

The phenotypic similarities observed between *hptB* and *retS* mutants led us to compare the transcriptome of these strains to reveal whether they have more target genes in common. We used *P. aeruginosa* microarrays that we engineered by spotting PCR products from around 4600 annotated *orfs* on the *P. aeruginosa* genome (*Experimental procedures*). The mRNAs were extracted from the PAK, PAKΔ*retS* and PAKΔ*hptB* strains grown in LB at 37°C in conditions that induce expression of the T3SS genes as previously described (Goodman *et al*., 2004; *Experimental procedures*). The cDNAs were synthesized and labelled using Cy3 or Cy5 (*Experimental procedures*). Gene expression levels in either PAKΔ*retS* or PAKΔ*hptB* mutants were directly compared with expression levels observed in the PAK strain (Table S2). Our data confirmed that the *pel* genes (*pelA* and *pelB*) were upregulated by about threefold in the PAKΔ*hptB* mutant and by about fourfold in the PAKΔ*retS* mutant (Table S2). One noticeable observation is that genes involved in T3SS are downregulated in the *hptB* mutant. The fold variation is from 2.3 (*PA1697/pscN*) to 7.4 (*exoY*). This is similar to that seen in the *retS* mutant, in which these genes have been previously shown to be downregulated (Goodman *et al*., 2004).

Our microarray analysis thus suggested that HptB positively controls expression of the *T3SS* genes. We confirmed this observation by introducing the plasmid pSB307, containing a transcriptional *exoS–lacZ* fusion (Bleves *et al*., 2005) into the PAK strain and the PAKΔ*hptB* mutant. Strains were grown in conditions that induce *T3SS* gene expression and β-galactosidase activity was measured at different growth stages. At all time points tested we observed downregulation of the reporter gene fusion by twofold in the *hptB* mutant as compared with PAK (Fig. 3B). It should be noticed that in the *retS* mutant the downregulation of the *exoS–lacZ* fusion is more pronounced (sevenfold) (Fig. 3B). By introducing the *hptB* gene *in trans* (pUCP*hptB*), wild-type expression levels could be readily restored in the *hptB* mutant (Fig. 3B). Our data confirmed that HptB, like RetS, positively regulates most of the *T3SS* genes.

Overall, we identified 19 genes whose expression varies significantly in the *hptB* mutant (Table S2) and all of them appeared to be also affected in the *retS* mutant (Table S2). It should be noted that the identity of the 127 genes whose expression varies in the *retS* mutant is consistent with previously published data (Goodman *et al*., 2004; Table S2). However, the transcription profiles of the *retS* and *hptB* mutants are not identical. Indeed, the entire HptB regulon is part of the RetS regulon, whereas the RetS regulon includes many genes that are not controlled by the HptB pathway. In particular, expression of the type VI secretion (T6SS) genes (Mougous *et al*., 2006; Filloux *et al*., 2008), which is significantly upregulated in the *retS* mutant (PA0078, PA0083-PA0087 and PA0089; Table S2), is not affected in the *hptB* mutant. We further checked that the T6SS-associated protein VgrG1 (PA0091) was not overproduced in the *hptB* mutant. Western blot analysis using antibodies directed against VgrG1 revealed that whereas it is produced in the *retS* mutant, it is hardly detectable either in the PAK strain or in the *hptB* mutant (Fig. S2). In conclusion, our analysis revealed that the HptB and RetS signalling pathways influence expression of a subset of common genes but are not fully overlapping.

### HptB overproduction does not compensate for the *retS* mutation

By using phenotypic assays, transcriptional fusions and transcriptome profiling, we have shown that at least two targets were common to RetS and HptB signalling pathways, the *pel* and *T3SS* genes. Because RetS is a hybrid sensor, HptB could have been the phosphorylation relay allowing activation of the RetS cognate RR. We investigated whether overproduction of HptB could restore a wild-type expression level for *pel* and *exoS* genes in a *retS* mutant. We tested the expression of the *pelA–lacZ* (Fig. 3A) and *exoS–lacZ* (Fig. 3B) transcriptional fusions as described earlier and noticed no variation in the level of β-galactosidase activity when comparing the *retS* mutant with the *retS* mutant overexpressing *hptB* (pUCP*hptB*). Failure of HptB overproduction to restore expression of *pel* and *exoS* genes in the *retS* strain was further confirmed using biofilm and Congo red assays (Fig. 2). However, overexpression of the *hptB* gene in an *hptB* mutant readily restored wild-type activity of the transcriptional fusions (Fig. 3A and B). Overall, these observations are not in favour of HptB being a cognate partner for RetS.

### HptB control is GacS/GacA-dependent

**Fig. 4:**
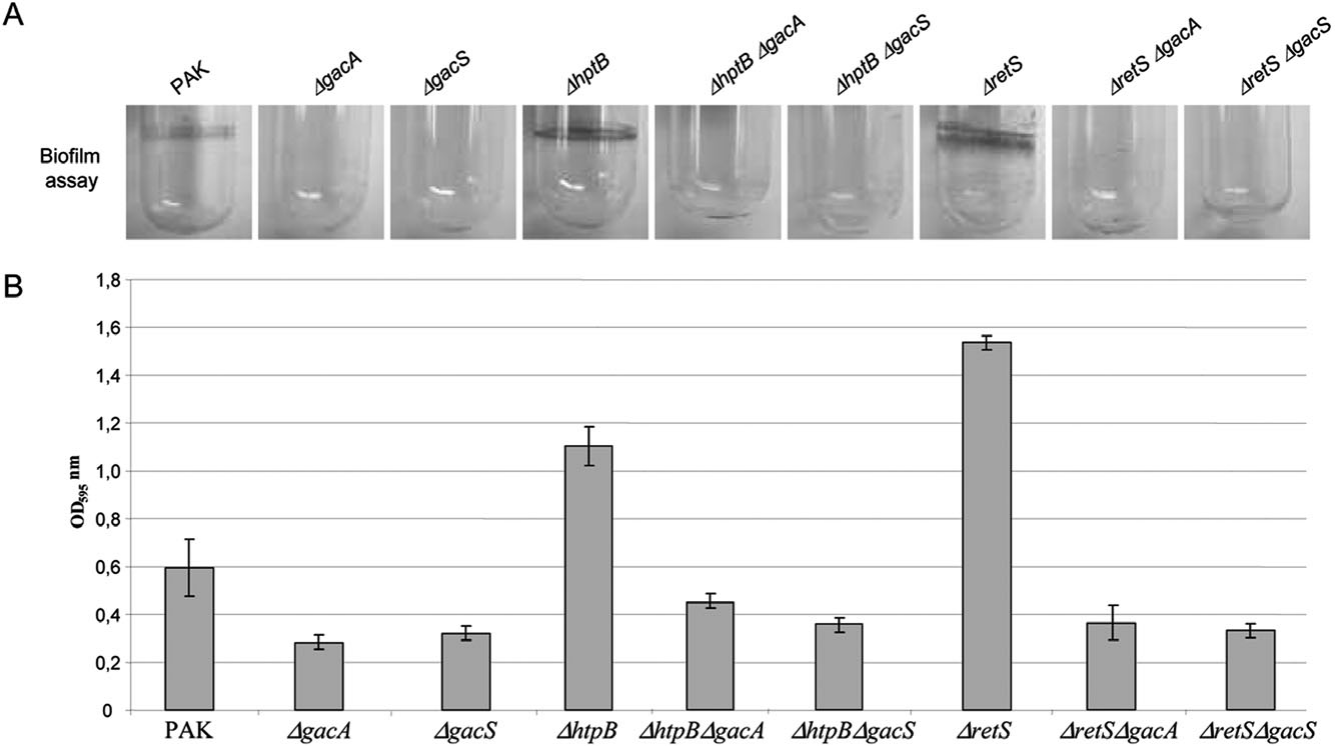
Effect of the *gacS* and *gacA* mutations on biofilm formation. The *gacS* or *gacA* mutation was introduced in PAK, PAKΔ*hptB* and PAKΔ*retS*. A. Glass tube assay showing biofilm formation. The name of the strain is indicated above each panel. B. Quantification of the crystal violet-stained adherence ring formed in the glass tube. Each experiment was repeated three times. The error bars indicate standard deviations. The name of strains used is indicated under each bar.

Our microarray analysis shows that all HptB target genes are also controlled by the RetS pathway. In a previous study (Goodman *et al*., 2004), it was shown that suppressor mutations of the *retS* phenotype could be found in genes involved in the GacS/GacA signalling pathway, suggesting that this pathway is required for the action of RetS on downstream target genes. We tested whether the HptB signalling pathway, like the RetS pathway, converges onto the GacS/GacA system. We speculated that if the HptB pathway was dependent on the Gac system, a *gacS* or *gacA* mutation should suppress the phenotype of an *hptB* mutant. Therefore, we engineered a *gacS* or *gacA* deletion into the PAKΔ*hptB* and PAKΔ*retS* strains (*Experimental procedures*). We compared the biofilm phenotypes of these strains with the PAK strain and the single *hptB* or *retS* mutant (Fig. 4). Whereas the *hptB* and *retS* mutants displayed a thicker crystal violet-stained ring in the microtitre plate assay compared with the wild-type (Fig. 4), the phenotype was abolished upon introduction of the *gacS* or *gacA* mutation in both the *hptB* and the *retS* mutants. We performed similar phenotypic observation using the Congo red staining assay (data not shown). Thus, the *hptB* mutation is suppressed by a secondary mutation in *gacS* or *gacA*, suggesting that HptB acts upstream of the Gac pathway or at the same level.

### HptB controls *rsmY* but not *rsmZ* gene expression

**Fig. 5:**
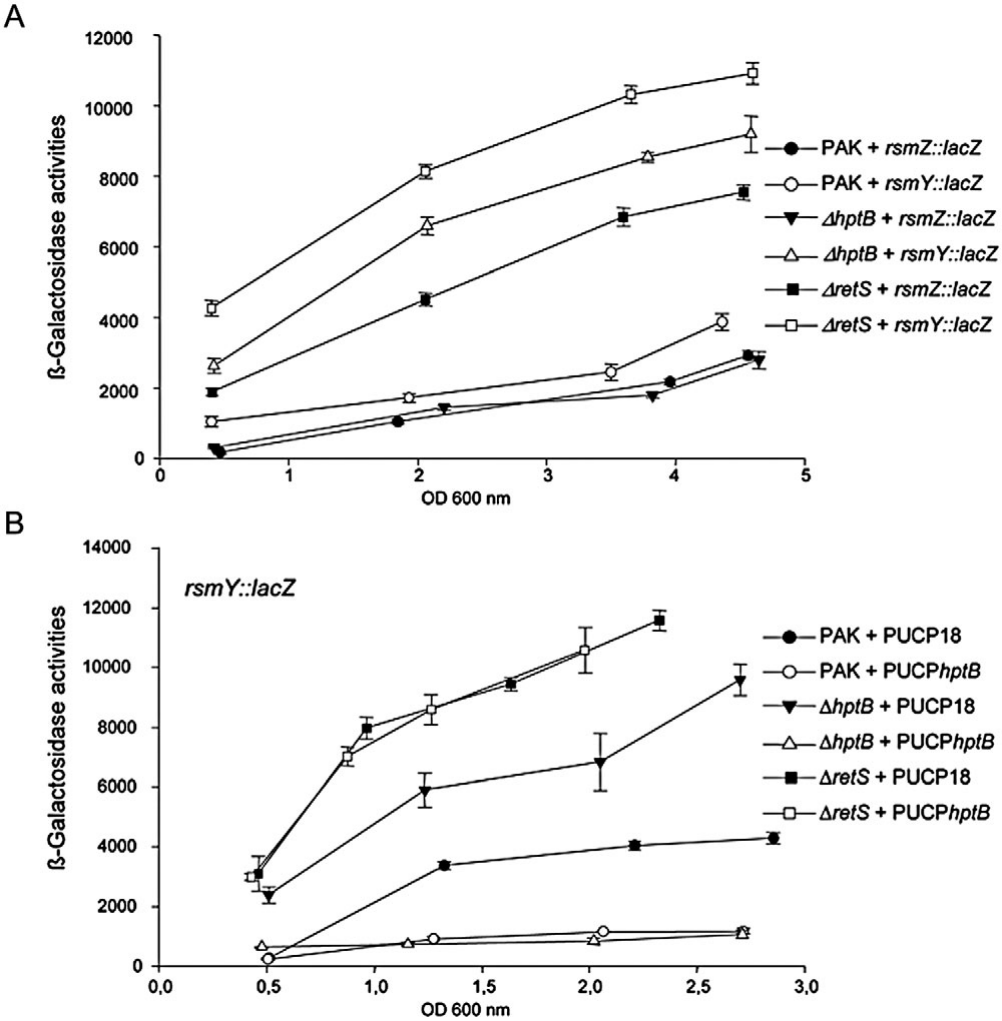
Expression of the *rsmY* and *rsmZ* genes in various *P. aeruginosa* strains. A. Activity of the *rsmY–lacZ* (open signs) and *rsmZ–lacZ* (filled signs) transcriptional fusion in PAK, PAKΔ*hptB* or PAKΔ*retS* strains was recorded at different growth stages. B. Activity of the *rsmY–lacZ* transcriptional fusions in PAK, PAKΔ*hptB* or PAKΔ*retS* strains, carrying pUCP18 (filled signs) or pUCP*hptB* (open signs), was recorded at different growth stages. β-Galactosidase activities are expressed in Miller units. Values are averages of at least three independent experiments.

**Fig. 6:**
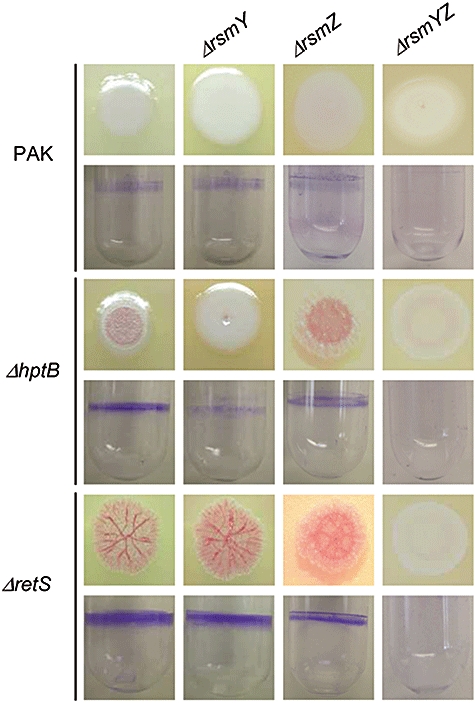
Influence of the *rsmY* and *rsmZ* mutations in PAK, PAKΔ*hptB* and PAKΔ*retS* strains for biofilm formation and exopolysaccharide production. For each row the name of the corresponding strain is indicated on the left. For each strain the upper row corresponds to the bacterial colony staining on Congo red-containing agar plates and the lower row to the glass tube assay showing biofilm formation. In each strain additional mutations in *rsmY*, *rsmZ* or *rsmY/rsmZ* have been introduced as indicated at the top of each column.

In previous studies, it was shown that the Gac pathway acts mainly through the modulation of sRNAs levels, namely RsmY and RsmZ (Kay *et al*., 2006; Brencic *et al*., 2009). We investigated the impact of HptB on *rsmY* and *rsmZ* regulation, and compared these results with the impact of RetS. We engineered *rsmY–lacZ* and *rsmZ–lacZ* transcriptional fusions (*Experimental procedures*), and in both cases we observed higher levels of β-galactosidase activities in the PAKΔ*retS* mutant as compared with PAK (3.8- to 4-fold) (Fig. 5A). The *rsmY–lacZ* fusion was also upregulated in the *hptB* mutant (Fig. 5A), but we could observe no effect of the *hptB* mutation on the expression of the *rsmZ–lacZ* transcriptional fusion (Fig. 5A). Importantly, introduction of *hptB in trans* in the *hptB* mutant abolished *rsmY* expression, whereas it had no effect when introduced into the *retS* mutant (Fig. 5B). We thus showed that RetS and HptB are independent signalling pathways, which act differently on the expression of the two small RNA-encoding genes, *rsmZ* and *rsmY*. This is a crucial observation, which reveals that the regulation of these sRNAs is partly different. We also observed that introduction of a *rsmY* mutation in the *hptB* mutant (*Experimental procedures*) suppresses the hyperbiofilm phenotype (Fig. 6) and confirmed that in this background the phenotype relies exclusively on RsmY but not on RsmZ. This is also clearly visible when looking at the phenotype of the *hptB/rsmY* mutant on Congo red-containing plates (Fig. 6). Instead, when the *rsmY* mutation is introduced in the *retS* mutant (Fig. 6), the hyperbiofilm phenotype is unaltered, suggesting that RsmZ could substitute for RsmY. Finally, only the simultaneous deletion of both *sRNA* genes is able to suppress *retS* phenotypes.

### RsmY and RsmZ contribution to the HptB or RetS signalling pathway

We have shown that RsmY is essential in the HptB signalling pathway, whereas in the RetS pathway either RsmY or RsmZ could be sufficient for proper signalling. In order to investigate this in more detail, we systematically engineered *rsmY*, *rsmZ* and *rsmY/rsmZ* deletion mutants in the PAK parental strain and the *hptB* and *retS* background. As expected the *rsmZ* mutation has no impact on Congo-Red staining or hyperbiofilm phenotypes when introduced in the *hptB* mutant (Fig. 6). Interestingly, when the *rsmZ* mutation was introduced in the *retS* genetic background, the Congo-Red and hyperbiofilm phenotypes were only slightly affected and were resembling the phenotypes of an *hptB* mutant. This observation makes sense, knowing that an *hptB* mutant overproduces RsmY but not RsmZ (Fig. 5). Finally, when a double mutation *rsmY/rsmZ* was introduced in *hptB* or the *retS* mutants the phenotypes observed for Congo-Red staining and biofilm were similar to the phenotype of strains, which do not overproduce any of the sRNAs (*gacA* mutant for example).

**Fig. 7:**
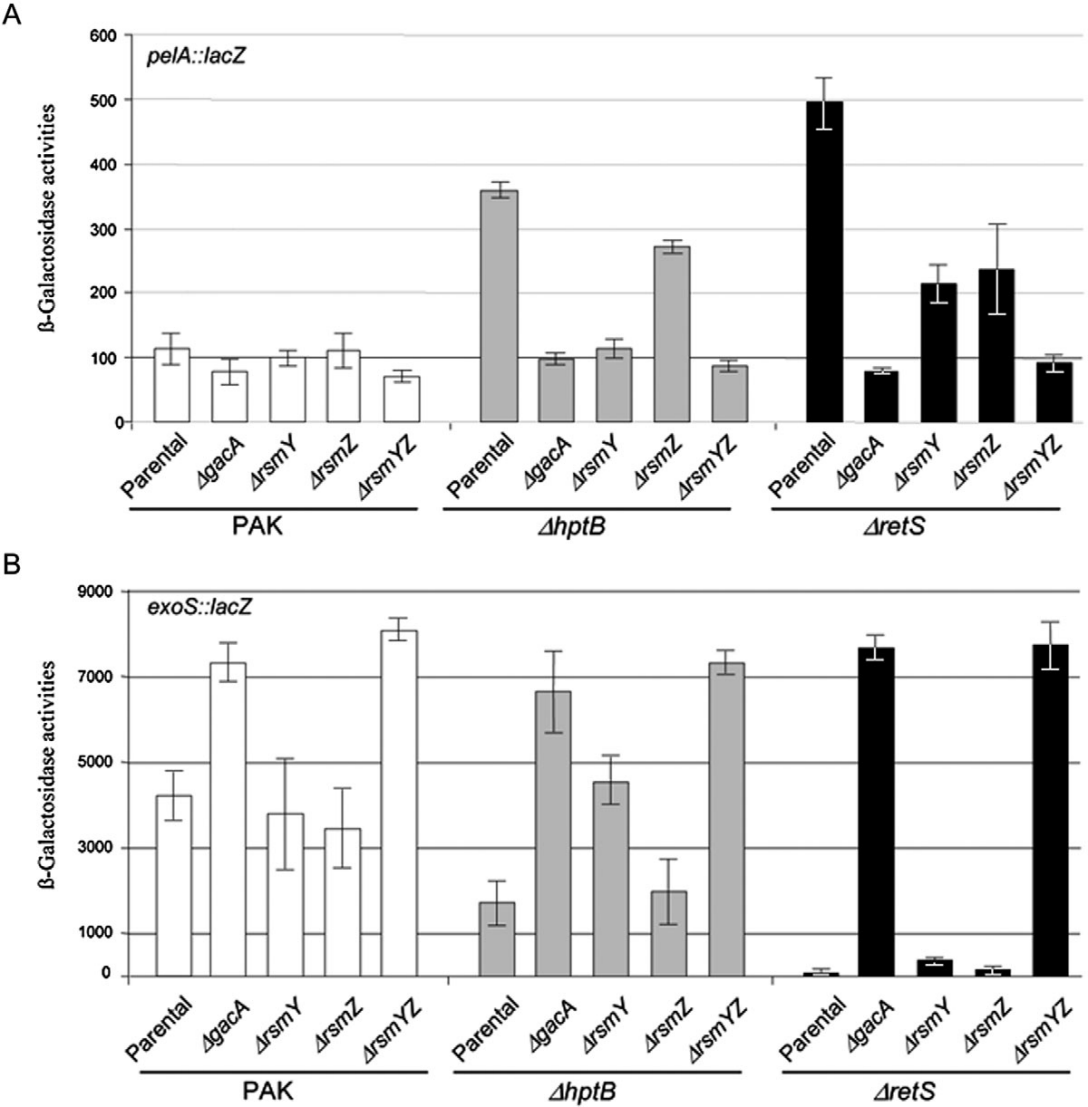
Expression of *lacZ* transcriptional fusion in PAK, PAKΔ*hptB* or PAKΔ*retS* strains. Activity was recorded after 4 h growth. A. Activity of the *pelA–lacZ* transcriptional fusion. B. Activity of the *exoS–lacZ* transcriptional fusion (carried on pSB307). White bars correspond to PAK, grey bars to PAKΔ*hptB* and black bars to PAKΔ*retS*. Each additional mutation, i.e. *gacA*, *rsmY*, *rsmZ* or *rsmYZ*, which was introduced in each of these strains, is indicated under the corresponding bar. β-Galactosidase activities are expressed in Miller units. Values are averages of at least three independent experiments.

We looked in more detail at the impact of these different mutations by analysing the expression of two transcriptional fusions, *pelA–lacZ* and *exoS–lacZ*, which are upregulated or downregulated, respectively, in an *hptB* or *retS* genetic background (Fig. 7A). Regarding the *pelA–lacZ* fusion, it is important to point out first that the influence of HptB on the expression of the transcriptional fusion is mostly RsmY-dependent. Indeed, introduction of the *rsmY* mutation in the *hptB* mutant drastically affects *pelA–lacZ* expression, whereas introduction of the *rsmZ* mutation has only little effect (Fig. 7A). In contrast, upon introduction of the *rsmY* or *rsmZ* mutation in the *retS* genetic background, the expression of the fusion is reduced by about twofold (Fig. 7A). Only when both mutations were introduced simultaneously was the level of *pelA–lacZ* transcription abolished and returned to the level observed in the PAK wild-type strain (Fig. 7A). This observation suggests that the control exerted by RsmY and RsmZ on *pelA* gene transcription is somehow additive. When analysing the fate of the *exoS–lacZ* transcriptional fusion in a *retS* background, we observed that either an *rsmY* or *rsmZ* mutation was not sufficient to allow expression of the fusion (Fig. 7B). Only introduction of both *rsmY* and *rsmZ* mutations into the *retS* background resulted in induction of the *exoS–lacZ* transcriptional fusion (Fig. 7B). This observation suggests that the control exerted by RsmY and RsmZ on *exoS* gene transcription is redundant and that deletion of either one of them in the *retS* background is sufficient to repress *exoS* expression. It is also important to note that the effect of the double mutation *rsmY/rsmZ* in the *retS* or *hptB* genetic background resulted in transcriptional levels of *pel–lacZ* and *exoS–lacZ* fusions similar to those observed in a *gacA* mutant (Fig. 7). This is good in agreement with the fact that GacA is the only known positive regulator for the expression of the sRNAs in *P. aeruginosa* (Brencic *et al*., 2009).

We also analysed, in these various genetic backgrounds, the level of production of the T6SS component VgrG1. We observed, using immunobloting and anti VgrG1, that, as expected from our microarray data, there is no induction of VgrG1 in the *hptB* mutant whereas high levels of VgrG1 are seen in the *retS* mutant. Upon introduction of the *rsmY* mutation in the *retS* mutant, the level of VgrG1 is slightly decreased (Fig. S2). However, when the *rsmZ* mutation is introduced in the *retS* mutant the level of VgrG1 is much more severely decreased. This observation makes sense since a *retS/rsmZ* mutant is likely to have a phenotype that is similar to the phenotype of an *hptB* mutant, which is what we observed (Fig. S2). Finally, when both *rsmY* and *rsmZ* are deleted in the *retS* mutant, the production of VgrG1 is abolished (Fig. S2).

### HptB acts through the PA3346/PA3347 regulatory components

**Fig. 8:**
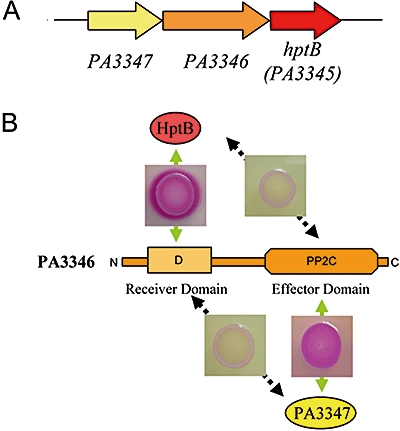
Interaction between HptB, PA3346 andPA3347. A. *PA3345* encodes Hpt and is clustered with *PA3346* and *PA3347* genes. B. Two-hybrid experiment showing interaction between HptB and the receiver domain of PA3346 and between the PP2C domain of PA3346 and PA3347 (shown with the insert and the red-stained colonies on Mc Conkey agar plates). Interaction between HptB and the PP2C domain of PA3346 or between the receiver domain of PA3346 and PA3347 are negative (shown with the insert and the white colonies on Mc Conkey agar plates). The N- and C-termini for PA3346 have been indicated.

**Fig. 9:**
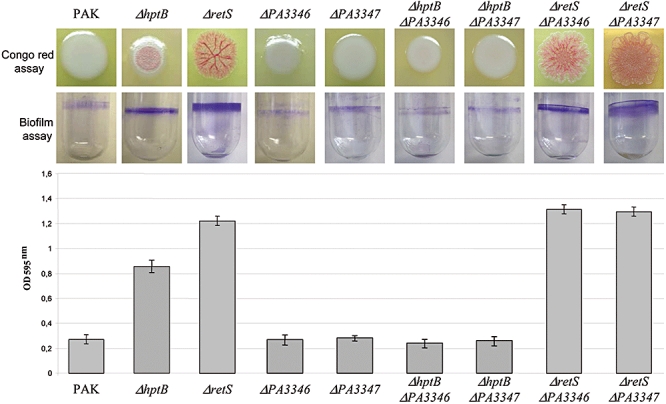
Influence of the *PA3346* and *PA3347* mutations in PAK, PAKΔ*hptB* and PAKΔ*retS* strains for biofilm formation and exopolysaccharide production (Congo red assay). For each column the name of the corresponding strain is indicated. For each strain the upper row corresponds to the bacterial colony staining on Congo red-containing agar plates, the middle row to the glass tube assay showing biofilm formation and the bottom row to the quantification of crystal violet staining as seen in the middle row.

**Fig. 10:**
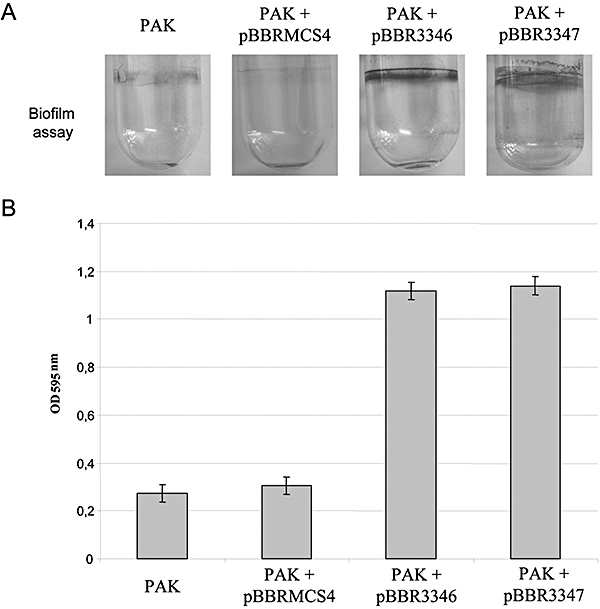
Effect of the overexpression of *PA3346* (pBBR3346) and *PA3347* (pBBR3347) on biofilm formation. The cloning vextor (pBBRMCS4) and the appropriate recombinant plasmids were introduced in PAK. A. Glass tube assay showing biofilm formation. The name of the strain is indicated above each panel. B. Quantification of the crystal violet-stained adherence ring formed in the glass tube. Each experiment was repeated three times. The error bars indicate standard deviations. The name of strains used is indicated under each bar.

The *hptB* gene is annotated as *PA3345*, and was shown to be organized as an operon together with the *PA3346* and *PA3347* genes (Hsu *et al*. 2008 and Fig. 8A). It was previously suggested that HptB interacts with the *PA3346* gene product (Hsu *et al*., 2008). This gene encodes an RR with an N-terminal phosphoryl-receiver domain and a C-terminal output domain belonging to the phosphatase 2C (PP2C) family (Delumeau *et al*., 2004). By using a two-hybrid system (*Experimental procedures*), we demonstrated that HptB directly interacts with the receiver domain of the PA3346 RR, but not with the PP2C domain (Fig. 8B). Furthermore, we showed by using the two-hybrid technique that the PP2C domain of the *PA3346* gene product, and not the receiver domain, interacts with the *PA3347* gene product, which encodes a putative anti-anti-σ factor (Fig. 8B). Since HptB, PA3346 and PA3347 appeared to belong to the same regulatory cascade, we analysed whether *PA3346* and *PA3347* deletion mutants (*Experimental procedures* and Table S1) displayed an *hptB* mutant phenotypes. Interestingly, neither the *PA3346* nor the *PA3347* mutant showed a hyper biofilm phenotype or increased staining on Congo red plates (Fig. 9). However, when a *PA3346* or *PA3347* mutation was introduced in the *hptB* mutant background, the *hptB* mutant phenotype readily disappeared (Fig. 9). In particular, these strains did not display the hyperbiofilm phenotype of the *hptB* mutant and behave like the PAK wild-type strain (Fig. 9). This is strongly suggesting that PA3346 and PA3347 are located downstream of HptB in the HptB signalling pathway. Moreover, when a *PA3346* or *PA3347* mutation was introduced in the *retS* mutant background, no changes in the *retS* phenotype were observed, suggesting that PA3346 and PA3347 are part of the HptB signalling pathway but do not intersect with the RetS signalling pathway (Fig. 9). We then tested the impact of *PA3346* and *PA3347* overexpression. Both genes were cloned in the pBBRMCS4 vector (*Experimental procedures* and Table S1) and the resulting recombinant plasmids (pBBR*3346* and pBBR*3347*) were introduced in the parental PAK strain. Overexpression of either *PA3346* or *PA3347* (Fig. 10) readily increased the level of biofilm formed by the PAK strain. Furthermore, we demonstrated that the gene targets whose expression is affected in the *hptB* mutant are similarly affected upon overexpression of *PA3346* or *PA3347*. Indeed, the *pelA–lacZ* transcriptional fusion is upregulated (Fig. S3A) in the PAK strain containing either pBBR*3346* and pBBR*3347*, while the *exoS–lacZ* fusion is downregulated in these same strains (Fig. S3B). This observation suggested that HptB antagonizes the activity of the couple PA3346/PA3347.

### PA3346/PA3347 control is GacS/GacA-dependent

We have shown previously that the HptB control is Gac-dependent. We thus checked whether PA3346/PA3347 activity is also dependent on a fully functional Gac system. The *PA3346*- and *PA3347*-containing plasmids were introduced in the *gacS* and *gacA* mutants and phenotypes were analysed. These two strains completely lost their ability to form hyperbiofilm (Fig. S4 and data not shown). Furthermore, the impact on *pelA–lacZ* or *exoS–lacZ* expression was totally abolished (Fig. S3A and B). Finally, since we have shown that the HptB signalling influences *rsmY* but not *rsmZ* gene expression, we assessed the impact of PA3346 and PA337 overproduction on RsmY and RsmZ levels. Whereas introduction of the pBBR3346 or pBBR3347 recombinant plasmids in the *rsmY–lacZ*-containing PAK strain resulted in high level of β-galactosidase activity (Fig. S5 and data not shown), no effect was observed when using the *rsmZ–lacZ*-containing strain (Fig. S5 and data not shown). These observations confirmed that PA3346 and PA3347 belong to the HptB signalling pathway.

## Discussion

The regulatory networks resulting in the production of virulence determinants by bacterial pathogens are increasingly complex. In *P. aeruginosa*, several regulatory devices including quorum sensing (Smith and Iglewski, 2003; Bleves *et al*., 2005), TCSs (Rodrigue *et al*., 2000) and chemotaxis (Garvis *et al*., 2009) have been involved in the chain of command determining the bacterial pathogenesis strategy.

Previous studies have depicted one such network involving the two-hybrid sensors, LadS and RetS, which antagonistically control biofilm formation and T3SS (Goodman *et al*., 2004; Ventre *et al*., 2006). Another study by Hsu and colleagues suggested that the HptB phosphorelay might be associated with RetS (Hsu *et al*., 2008). In the present study, we showed that the *hptB* and the *retS* mutants are hyperbiofilm formers and that in both cases the hyperbiofilm phenotype is linked to overexpression of the *pel* genes. We also showed that, as for a *retS* mutant, *T3SS* genes are downregulated in the *hptB* mutant. From this perspective, it seems that RetS and HptB signalling pathways share target genes and may be part of the same signalling pathway.

However, we collected information that suggests that RetS and HptB are not cognate partners. One observation is that the biofilm formed by the *hptB* mutant, even though thicker as compared with the parental PAK strain, is not as thick as the *retS* mutant biofilm (Fig. 1B and Fig. 6). Furthermore, microarray analysis revealed that only a subset of the RetS target genes are HptB targets. For example, the *T6SS* genes (Mougous *et al*., 2006; Filloux *et al*., 2008) are upregulated in the *retS* mutant, but remained unaffected in the *hptB* mutant. Finally, RetS forms heterodimers with GacS, inhibits GacS autophoshorylation and prevents activation of the GacA RR (Goodman *et al*., 2009). It was also shown that RetS phosphorelay domains are not required for its function, suggesting that RetS is not likely to act through a phosphorelay such as HptB, but rather exclusively through GacS heterodimerization (Goodman *et al*., 2009).

In response to GacS, GacA controls levels of sRNA, which in turn relieve translational repression by the RsmA protein on several mRNAs (Pessi *et al*., 2001; Brencic and Lory, 2009). In *P. aeruginosa*, RsmA could be out-titrated by high levels of the sRNAs, RsmY and RsmZ (Kay *et al*., 2006; Brencic *et al*., 2009). The GacS/GacA regulation is most exclusively exerted through the transcriptional control of *rsmY* and *rsmZ* genes expression (Brencic *et al*., 2009), although sRNAs may exist that are regulated by GacA in an indirect manner (Livny *et al*., 2006; González *et al*., 2008).

In our study we confirmed that RetS negatively controls *rsmY* and *rsmZ* gene expression, in a GacA-dependent manner. We analysed whether HptB constitutes another branch of the Gac/Rsm signalling pathway. We hypothesized that if GacA is located downstream of HptB in the signalling cascade, the lack of GacA in the *hptB* mutant should abolish the hyperbiofilm phenotype induced by the *hptB* mutation, which is what we observed (Fig. 4). We also showed that the *hptB* phenotype could be suppressed by a mutation in *gacS*, suggesting that as for RetS the control by HptB on the target genes is likely to be indirect and should at some stage go through GacS/GacA or at least required this system as co-activator.

As part of the Gac signalling pathway it should be expected that HptB controls expression of the *rsmZ* and *rsmY* genes, since GacA is known to bind directly to the promoter regions of *rsmY* and *rsmZ*. However, in the *hptB* mutant, we found that in contrast to the *retS* mutant, only the *rsmY–lacZ* fusion is upregulated. These are clear evidence that regulation of *rsmZ* and *rsmY* is different with *rsmY* expression controlled by both RetS and HptB, whereas *rsmZ* is exclusively controlled by RetS. One possible explanation for this original observation is that additional regulatory components are involved in *rsmY* and *rsmZ* gene expression. It is reported that both regions upstream of the *rsmY* and *rsmZ* promoters contain a GacA binding site (Kay *et al*., 2006; Brencic *et al*., 2009). However, these regions also display clear differences in length and structure. For example, it was recently shown that two members of the H-NS family of global regulators, MvaT and MvaU, could bind the *rsmZ* but not the *rsmY* promoter (Brencic *et al*., 2009).

In addition to the observation that sRNAs control is significantly different depending on whether it is operated from the RetS or HptB signalling pathway, we also observed that RsmY and RsmZ exert their effect on target genes either in an additive or redundant/compensatory manner. This was quite surprising since in the case of the *P. aeruginosa* RsmY and RsmZ, it was admitted that, as with the RsmY/RsmZ/RsmX of *P. fluorescens* (Kay *et al*., 2005), the sRNAs are functionally redundant.

The functional redundancy of sRNAs may, however, be a matter of debate. For example, in *V. cholerae* the four sRNAs, Qrr1–4, are functionally redundant and all four *sRNAs* must be deleted to see an effect on *hapR* mRNA stability (Lenz *et al*., 2004). This phenomenon was described as a gene dosage compensation mechanism, since a deletion in one of the *qrr* genes could be compensated for by an increase in the levels of remaining Qrrs (Svenningsen *et al*., 2009). By contrast, in *Vibrio harveyi* the five homologous Qrrs are not redundant, but act additively to translate signal into a precise gradient of LuxR (HapR homologue) (Tu and Bassler, 2007). In conclusion, in *V. cholerae*, expression of only one of the four sRNAs will be sufficient to target all *hapR* mRNA present within the cell, whereas in *V. harveyi*, in order to reach sufficient concentration of sRNAs and destabilize all *luxR* mRNA, all Qrrs should be expressed.

In the present study, we observed that the additive and redundant mechanisms seen in *V. harveyi* and *V. cholerae*, respectively, are combined in *P. aeruginosa*. In the case of *pel* gene expression, whose expression is high in a *retS* background, we noticed an obvious reduction in expression if an additional *gacA* mutation or a double *rsmY/rsmZ* mutation is introduced (Fig. 7). However, if only the *rsmY* or *rsmZ* mutation is introduced, in both cases the level of expression is only decreased by half. The impact of the *rsm* mutations is additive since only the double *rsmY/rsmZ* mutant mimics the phenotype of a *gacA* mutant. In the case of the *exoS* gene (representative for *T3SS* genes), we observed that expression is quasi null in the *retS* background, but dramatically relieved when the *gacA* mutation or the double *rsmY/rsmZ* mutation was further introduced. However, in this case, a single mutation in either *rsmY* or *rsmZ* had no impact on *exoS*, whose expression remains totally repressed as it is in the *retS* mutant (Fig. 7). Thus for *exoS* gene expression, in contrast to *pel* genes, the impact of sRNAs is redundant.

In summary, we have shown in this work that the phosphorelay involving HptB plays a specific role in exclusively controlling expression of the *rsmY* gene, providing further knowledge on the regulatory and signalling networks that control *P. aeruginosa* virulence and biofilm formation. This control is dependent on GacS/GacA, which normally acts both on *rsmY* and *rsmZ* expression. GacA is located at the end of a complex network of signalling pathways, which involve GacS, LadS, RetS and HptB. We suggest that HptB and RetS are two distinct signalling pathways both intersecting with the GacS/GacA system but through different mechanisms. Whereas RetS interferes directly with GacS autophosphorylation (Goodman *et al*., 2009), the pathway leading from HptB to the regulation of *rsmY* via the Gac pathway is still not clearly established.

**Fig. 11:**
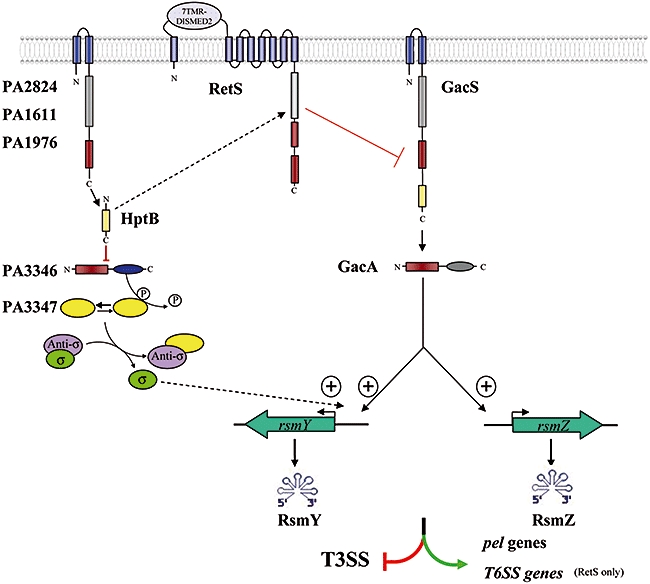
Model for the HptB regulatory network. HptB has a negative impact on PA3346 activity. In the absence of HptB, PA3346 dephosphorylates the putative anti-anti-σ factor PA3347 (yellow) through the activity of its PP2C domain (in blue). Dephosphorylated PA3347 could bind a putative anti-σ factor (purple), which allows the release of a yet uncharacterized σ factor (green). This σ factor may have a specific impact on *rsmY* gene expression, but not on *rsmZ* gene expression. The controlled expression of *rsmY* through the HptB/PA3346/PA3347 cascade is still GacA-dependent, suggesting GacA synergistically acts on the *rsmY* promoter together with the unknown σ factor. Overproduction of RsmY alone (through the PA3346/PA3347 pathway) results in overexpression of *pel* genes and repression of *T3SS* genes but not in overexpression of the T6SS genes, which are specifically controlled through the RetS pathway. The rest of the model integrates previous published data. The activity of RsmY and RsmZ is through RsmA titration, which is not represented in the figure. The RetS control through interference with the GacS activity was previously published (Goodman *et al*., 2009). The potential role of three hybrid sensors, PA2824, PA1611 or PA1976 on HptB activation and the retro-transfer of phosphate from HptB onto RetS comes also from previously published data (Hsu *et al*., 2008).

The novel HptB pathway might involve intermediate components such as the PA3346/PA3347 proteins (Hsu *et al*., 2008). In this study, we confirmed that HptB interacts with PA3346 using the two-hybrid system. PA3346 was proposed to encode an RR with a phosphatase 2C domain (PP2C) as output domain (Hsu *et al*., 2008). Hsu and collaborators showed that PA3346 could dephosphorylate PA3347, which encodes a protein with similarity to anti-anti-σ factor (Hsu *et al*., 2008). We showed that the PP2C domain of PA3346 is able to interact with PA3347 using the two-hybrid system. The PA3346/PA3347 cascade could resemble the RsbU/RsbV cascade in *Bacillus subtilis* (Delumeau *et al*., 2004), in which dephosphorylation of the anti-anti-σ factor RsbV by the phosphatase RsbU resulted in the capture of the anti-σ factor RsbW by RsbV and the release of the σ^B^ factor. Finally, we showed that, as for HptB, the activity of PA3346/PA3347 impacts *rsmY* but not *rsmZ* gene expression (Fig. S5). A putative model summarizing these features is presented in Fig. 11.

The way HptB influences differentially the activity of the *rsmY* and *rsmZ* promoters is a first level of complexity that still needs to be understood. The most likely explanation is that the *rsmY* promoter may contain a putative binding site for the unknown σ factor, which is released when the HptB pathway is activated, whereas the *rsmZ* promoter does not contain this alternative σ factor-binding site. Another level of complexity is given by a non-uniform control (additive or redundant) of the sRNAs, RsmY and RsmZ, on different target genes, although both sRNAs function by titrating RsmA. Here, the subtle relative stoichiometry between sRNAs, RsmA and target mRNAs is likely to be a key to understand this mechanism and might involve complex regulatory loops, as those proposed in the case of *V. cholerae* and *V. harveyi* sRNAs control (Svenningsen *et al*., 2008; Tu *et al*., 2008).

It seems clear now that the role of sRNAs is central to allowing for a quick and subtle bacterial response and adaptation to fast and tiny changes in environmental conditions. The complexity and connectivity of the regulatory circuits involved are yet far to be understood and we are pursuing the reconstruction of these networks by using systematic genetic screens, two-hybrid and *in vitro* phosphorylation assays to identify cognate partners and cross-talk between these different signalling pathways. We are currently working on the HptB signalling pathway and aim to identify all the components lying between HptB and the *rsmY* gene. In particular, we will try to identify the putative σ factor within the cascade involving the PA3347 anti-anti-σ factor.

## Experimental procedures

### Bacterial strains, plasmids and growth conditions

Bacterial strains and plasmids are described in Table S1. The nucleotide sequence of oligonucleotides used is given in Table S3. For engineering the *hptB* deletion mutant, the upstream and downstream sequences (approximately 500 bp) were amplified from PAK genomic DNA using the pair of primers BupA/BloA and BupB/BloB respectively. The PCR products were digested with EcoRI and cloned in tandem into pCR2.1. The linked DNA fragment was digested with XbaI and SpeI and cloned in suicide vector pKNG101, yielding pKNGΔ*hptB*. The suicide plasmid was introduced in PAK and deletion on the chromosome selected as previously described (Kaniga *et al*., 1991). For engineering the *gacS*, *gacA, rsmY, rsmZ, PA3346* and *PA3347* deletion mutants, the upstream and downstream regions (650 bp) of each gene were amplified from PAK genomic DNA using a specific couple of primers. PCR products were digested by BamHI and EcoRI for the upstream fragment and EcoRI and SpeI for the downstream fragment. Construction of suicide vector and allelic replacement is as above. The *hptB/gacA, hptB/gacS, hptB/rsmY, hptB/rsmZ, retS/gacA, retS/gacS, retS/rsmY* and *retS/rsmZ* double mutants were constructed by introducing the *gacS*, *gacA, rsmY* and *rsmZ* gene deletion in the PAKΔ*hptB* and PAKΔ*retS* strains. The *hptB/rsmYZ* and *retS/rsmYZ* triple mutants were constructed by introducing the *rsmZ* gene deletion in the PAKΔ*hptB/ΔrsmY* and PAKΔ*retS/ΔrsmY* strains. The *hptB/pelB* double mutant was constructed by using the pKNGmamb3063 suicide vector (Vasseur *et al*., 2005) in order to delete the *pelB* gene in the PAKΔ*hptB* strain.

The *hptB* gene was amplified by PCR from PAK genomic DNA using the primers Bup and Bdown. The PCR product was modified using a DNA blunting kit (Takara) and cloned into pUCP18, yielding pUCP*hptB*.

The PA3346 and PA3347 genes were amplified by PCR from PAK genomic DNA using the primers. The PCR products were digested by EcoRI and BamHI and cloned into pBBRMCS4, yielding, respectively, to pBBR3346 and pBBR3347. The *hptB/PA3346*, *hptB/PA3347*, *retS/PA3346* and *retS/PA3347* double mutants were constructed by introducing the *PA3346* and *PA3347* gene deletion in the PAKΔ*hptB* and PAKΔ*retS* strains.

The *rsmZ* and *rsmY* promoter regions were amplified from the PAK genome by using the oligonucleotides couple PrsmZ1/PrsmZ2 and PrsmY1/PrsmY2 respectively. The upstream primers PrsmZ1 and PrsmY1 contain an EcoRI site whereas the PrsmZ2 and PrsmY2 primers contain a KpnI site. The EcoRI/KpnI digested PCR products were cloned into pMP220 to yield *rsmZ–* and *rsmY–lacZ* transcriptional fusions.

Plasmids were introduced into *P. aeruginosa* by electroporation (Smith and Iglewski, 1989) or triparental mating using the conjugative properties of pRK2013 (Figurski and Helinski, 1979). The transformants were selected on *Pseudomonas* isolation agar. Antibiotics were used at the following concentrations for *E. coli*: 50 µg ml^−1^ ampicillin, 50 µg ml^−1^ streptomycin, 15 µg ml^−1^ tetracycline. For *P. aeruginosa*, 500 µg ml^−1^ carbenicillin, 200 µg ml^−1^ tetracycline and 2000 µg ml^−1^ streptomycin were used. Bacteria were grown in LB or M63 minimal medium supplemented with 0.2% glucose, 1 mM MgCl_2_ and 0.5% casamino acids.

### Adherence assays on inert surface

The *P. aeruginosa* adherence assay was performed in 24-wells polystyrene microtitre dishes (Vallet *et al*., 2001), or by inoculating glass tubes containing 1 ml of medium. Biofilm formation was visualized by using the crystal violet staining procedure and quantified after 5 h of incubation at 30°C (Vallet *et al*., 2001).

### *Pseudomonas aeruginosa* microarrays

A total of 4620 PCR products corresponding to 83% of the *P. aeruginosa* PAO1 genome have been spotted on glass slides. Most of the PCR products were obtained by using the PAO1 gene collection (Labaer *et al*., 2004), which allows amplification of all genes by using a single couple of primers (L1R1/L2R2, Table S3), flanking each *orf* cloned in the Gateway vector used for constructing this library. The amplification of the different *orfs* was done on bacterial colonies or on plasmidic DNA purified using a NucleoSpinR Multi-96 Plus Plasmid kit (Macherey-Nagel). For some genes longer than 3700 bp or not available in the library, amplicons were obtained by using *P. aeruginosa* genomic DNA as matrix and using specific primers yielding DNA fragments of about 500 bp. That was the case for the genes annotated PA0041, PA0690, PA0994, PA0844, PA1868, PA2462, PA3724, PA4084 and PA4541. All PCR products were purified using a QIAquickTM 96-well PCR purification kit (QIAgen). Microarrays were printed at the DNA microarray production platform at Sophia-Antipolis (IPMC-CNRS) using a ChipWriter Proarrayer (Bio-Rad). Each PCR product was spotted 4 times on commercial UltraGAPSTM slides of 24 × 60 mm (Corning Incorporated, MA, USA). DNA binding to the GAPS-coated surface was enhanced by UV cross-linking. Before performing the hybridization, microarrays were pre-hybridized with a BSA solution in order to block the empty surface of the slide, which helps to decrease non-specific hybridization.

### RNA isolation procedure

Overnight bacterial cultures were diluted in LB, containing 5 mM EGTA and 20 mM MgCl_2_, to 0.1 unit of OD_600_. The bacteria were grown under agitation at 37°C and were harvested during exponential phase (0.6 unit of OD_600_), by centrifugation at 4°C. The samples were quickly processed to prepare RNA using the ‘SV Total RNA Isolation System’ from Promega. The DNase I digestion step was carried out twice in order to diminish the quantity of contaminating DNA. The integrity of the RNA preparations was checked after electrophoresis on agarose gel. The absence of DNA contamination was verified by performing PCR reaction. The RNA was further used to prepare cDNA.

### cDNA synthesis and hybridization

Probes were generated by using the ChipShot Direct Labeling and Clean-Up System kits (Promega). Briefly, 10 µg of RNA was first hybridized with hexameric primers. Then dNTPs (330 nM each) were added together with 1 mM of Cy3 or Cy5 (Amersham) and 200 units of ChipShot Reverse transcriptase. The mixture was incubated 2 h at 42°C and the reaction stopped by a 15 min incubation at 37°C with RNAse. Unincorporated nucleotides were removed by using the ChipShot Direct Labeling and Clean-Up System (Promega). Labelled Cy3 or Cy5 cDNA were dried by using a speedVac and resuspended in 50 µl of ‘Dig Easy’ solution (Roche). The labelled cDNA was denatured upon a 5 min incubation at 95°C and used for microarray hybridization, 16 h at 42°C in a hybridization oven. The arrays were washed as follows: 2 times with 2× SSPE buffer, 0.1% SDS warmed at 62°C, 1 time with 0.5× SSPE buffer and 1 time with 0.1× SSPE buffer. The arrays were dried and scanned for data acquisition.

### Quantification and analysis of the microarrays

Data from scanned microarrays were acquired by using Genepix Pro 6 software (Molecular Devices). The data were subsequently processed by using Acuity software (Molecular Devices). All data were normalized by performing a Lowess regression and filtered to remove genes, which presented a weak expression in both conditions (signal noise ratio > 2). Two criteria (> threefold change and Student's *t*-test, *P* ≤ 0.05) were used to determine significant changes. Each microarray experiment was performed in triplicate with independent bacterial cultures.

### Measurements of β-galactosidase activity

Strains carrying the *lacZ* transcriptional fusions were grown in LB with agitation at 37°C. The bacterial cells were collected by centrifugation at different growth times. The β-galactosidase activity was measured using the method of Miller (Sambrook *et al*., 1989). Experiments with strains carrying the *exoS–lacZ* fusion carried on pSB307 were performed similarly except that EGTA (5 mM) and MgCl_2_ (20 mM) were added in the growth medium in order to induce *T3SS* genes expression.

### Congo red assay

Tryptone (10 g l^−1^) agar (1%) plates were supplemented with Congo red (40 µg ml^−1^) and Coomassie brilliant blue dyes (20 µg ml^−1^). Bacteria were inoculated on the surface of the plates with a toothpick and grown at 30°C. The colony morphology and staining were recorded after 2 days.

### Production of polyclonal VgrG1 antibodies

A V5-hexahistidine (V5H6) tag was added to the C-terminus of VgrG1 according to the Gateway® technology and using the original entry clone from the PAO1 *orf* collection (Labaer *et al*., 2004) and the pET-Dest42 destination vector, to give the recombinant vector pET*vgrG1*. The resulting construct was transferred into *E. coli* BL21 (DE3). The recombinant protein was overproduced in 100 ml of LB broth supplemented with 1 mM IPTG during 3 h at 37°C. Bacteria collected by centrifugation were sonicated in 5 ml of cold 10 mM Tris/HCl pH 8.0 containing protease inhibitor cocktail (Roche) and disrupted by sonication. Lysed bacteria were centrifuged at 4000 *g* and insoluble fraction enriched in inclusion bodies containing VgrG1 were washed twice in the sonication buffer and solubilized in Urea 8 M. Lysates were centrifuged at 40 000 *g*, and soluble fraction containing recombinant VgrG1 was applied on His Trap Hp column for purification (GE healthcare). About 1.5 mg of protein was purified and immunization protocols were performed at Eurogentec. Two rabbits were inoculated with 200 mg of VgrG1 protein, followed by three boosters spaced by 15 days, 1 month and 2 months. After that period, rabbits were sacrificed, and sera were verified for their specificity to VgrG1.

### Sodium dodecyl sulphate-polyacrylamide gel electrophoresis and immunoblotting

Bacterial cell pellets were resuspended in loading buffer (Laemmli, 1970). The samples were boiled and separated on SDS gels containing 10% acrylamide and blotted onto nitrocellulose membranes. After 30 min of saturation in Tris-buffered saline (TBS) (0.1 M Tris, 0.1 M NaCl, pH 7.5), 0.05% Tween 20 and 5% skim milk, the membrane was incubated for 1 h with anti-VgrG1 diluted 1:500; washed three times with TBS-0.05% Tween 20; incubated for 45 min with anti-rabbit immunoglobulin G (IgG) antibodies (Sigma) diluted 1:5000; washed three times with TBS-0.05% Tween 20; and then revealed with a Super Signal Chemiluminescence system (Pierce).

### The bacterial two-hybrid assay

DNA fragments encoding protein domains of interest were cloned at the 3′ end of genes encoding the two fragments of adenylate cycles carried on the pKT25 and pUT18c as described by Karimova and colleagues (2000). The DNA regions encoding the HptB protein, the receiver domain or PCR of the PP2C domain of PA3346 and the PA3347 protein were amplified by using PAK genomic DNA. PCR product on HptB and of the PP2C domain of PA3346 were digested by XbaI and KpnI and cloned into pKT25, yielding, respectively, to pKT25-*hptB* and pKT25-*PP2*. PCR product on PA3347 and of the receiver domain of PA3346 domain were digested by XbaI and EcoRI and cloned into pUT18C, yielding, respectively, to pUT18C-3347 and pUT18C-3346D. An adenylate cyclase deficient *E. coli* strain, DHM1, was used to screen for positive interactions. DHM1 competent cells were transformed simultaneously with pKT25 and pUT18c derivatives and transformants were selected on agar plates supplemented with ampicillin (100 µg ml^−1^) and kanamycin (50 µg ml^−1^). Single colonies were patched on MacConkey medium (Difco) supplemented with IPTG (1 mM) and maltose (1%). Positive interactions were identified as red colonies after 24 h incubation at 30°C.
