# Supplementary material for: Regulatory RNAs and the HptB/RetS signalling pathways fine-tune Pseudomonas aeruginosa pathogenesis
Source: Mol Microbiol. 2010 Apr 12;76(6):1427–43. doi: 10.1111/j.1365-2958.2010.07146.x (PMC2904497; doi:10.1111/j.1365-2958.2010.07146.x)
Supplement: Supplementary file 1 [file mmi0076-1427-SD1.pdf]

SUPPORTING INFORMATION

Figure S1

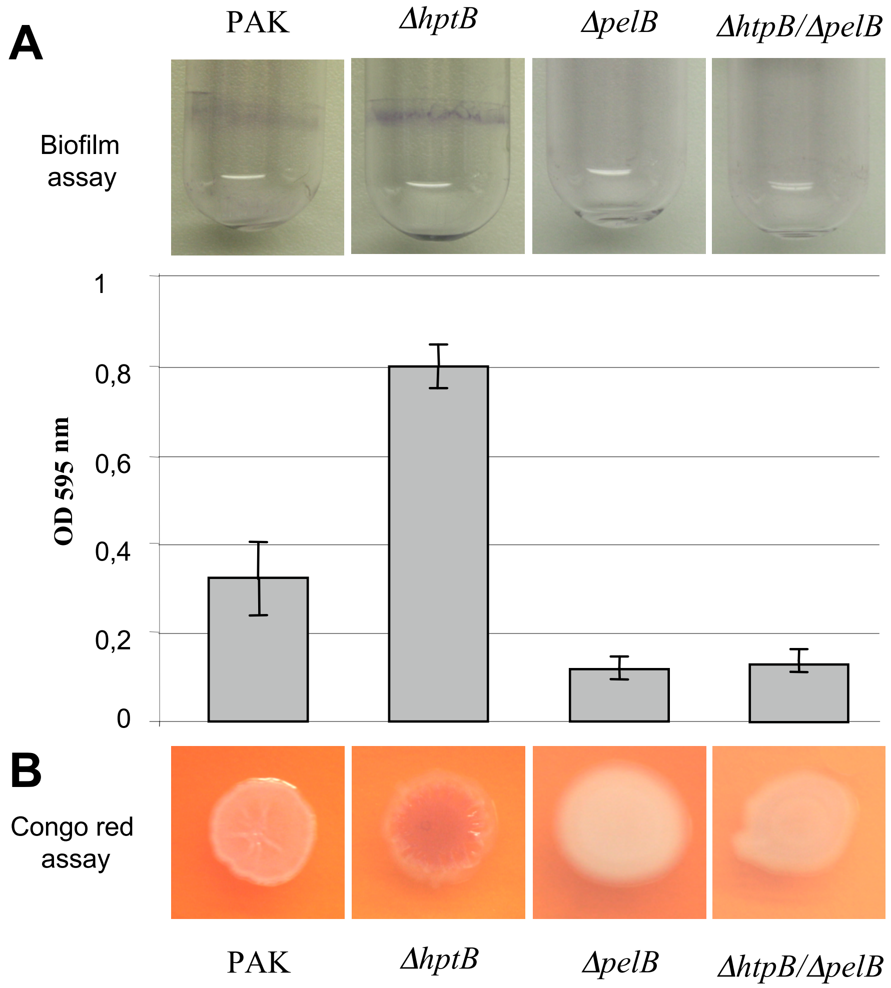

Fig. S1. Effect of *pelB* deletion gene on *htpB* phenotypes.

A. Glass tube assay showing biofilm formation (upper part). Quantification of the crystal violet-stained adherence ring formed in the glass tube (lower part). Each experiment was repeated three times. The error bars indicate standard deviations. The name of the tested strain is indicated above each panel.

B. Bacterial colony staining on Congo Red-containing agar plates. The name of strains used is indicated under each panel.

Figure S2

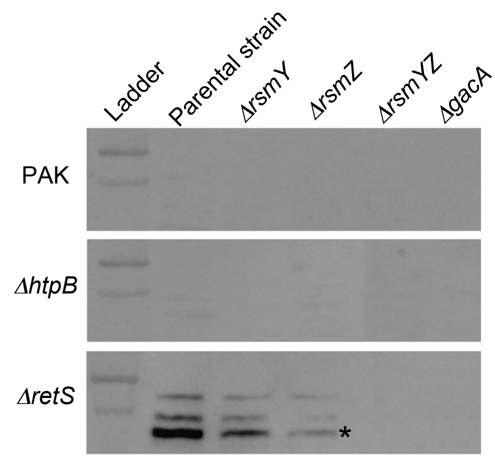

Fig. S2. Production of the type VI secretion component VgrG1. Immunodetection of VgrG1 (indicated by an asterisk) on whole cell extracts of strains PAK, PAKΔ*hptB* and PAKΔ*retS* indicated as parental strain). VgrG1 production was also tested in each parental strain carrying an additional *rsmY*, *rsmZ*, *rsmYZ* or *gacA* mutation.

Figure S3

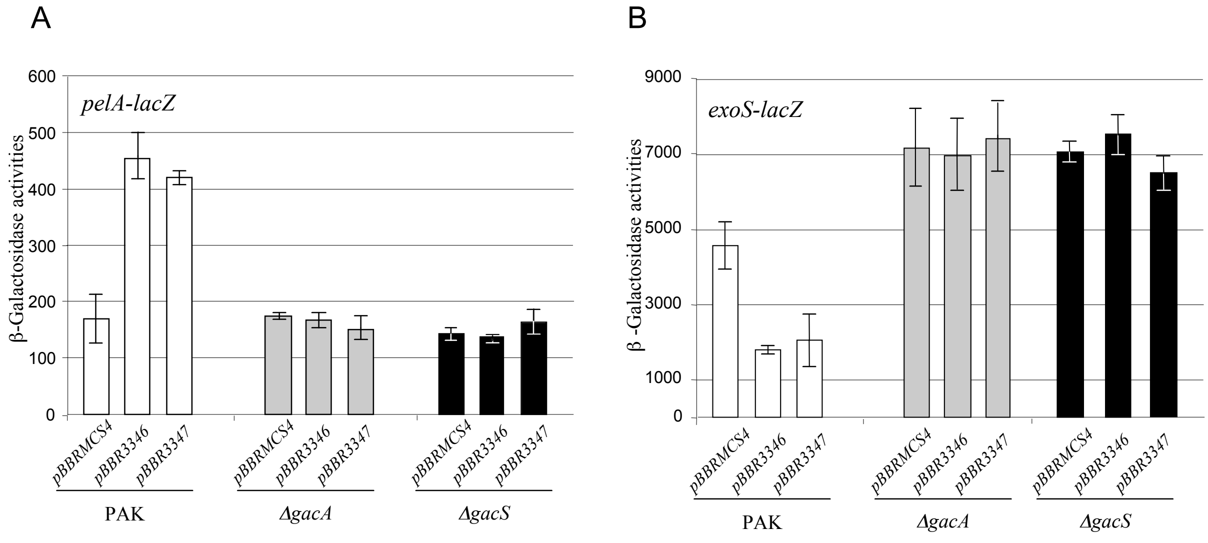

Fig. S3. Expression of *lacZ* transcriptional fusion in PAK, PAKΔ*gacA* or PAKΔ*gacS* strains overexpressing *PA3346* (pBBR3346) or *PA3347* (pBBR3347) or containing the cloning vector pBBRMCS4. Activity was recorded after 4 hours growth.

A. Activity of the *pelA-lacZ* transcriptional fusion.

B. Activity of the *exoS-lacZ* transcriptional fusion (carried on pSB307). White bars correspond to PAK, grey bars to PAKΔ*gacA* and black bars to PAKΔ*gacS*. The plasmid contained by each of these strains is indicated under the corresponding bar.

β-galactosidase activities are expressed in Miller units. Values are averages of at least three independent experiments.

Figure S4

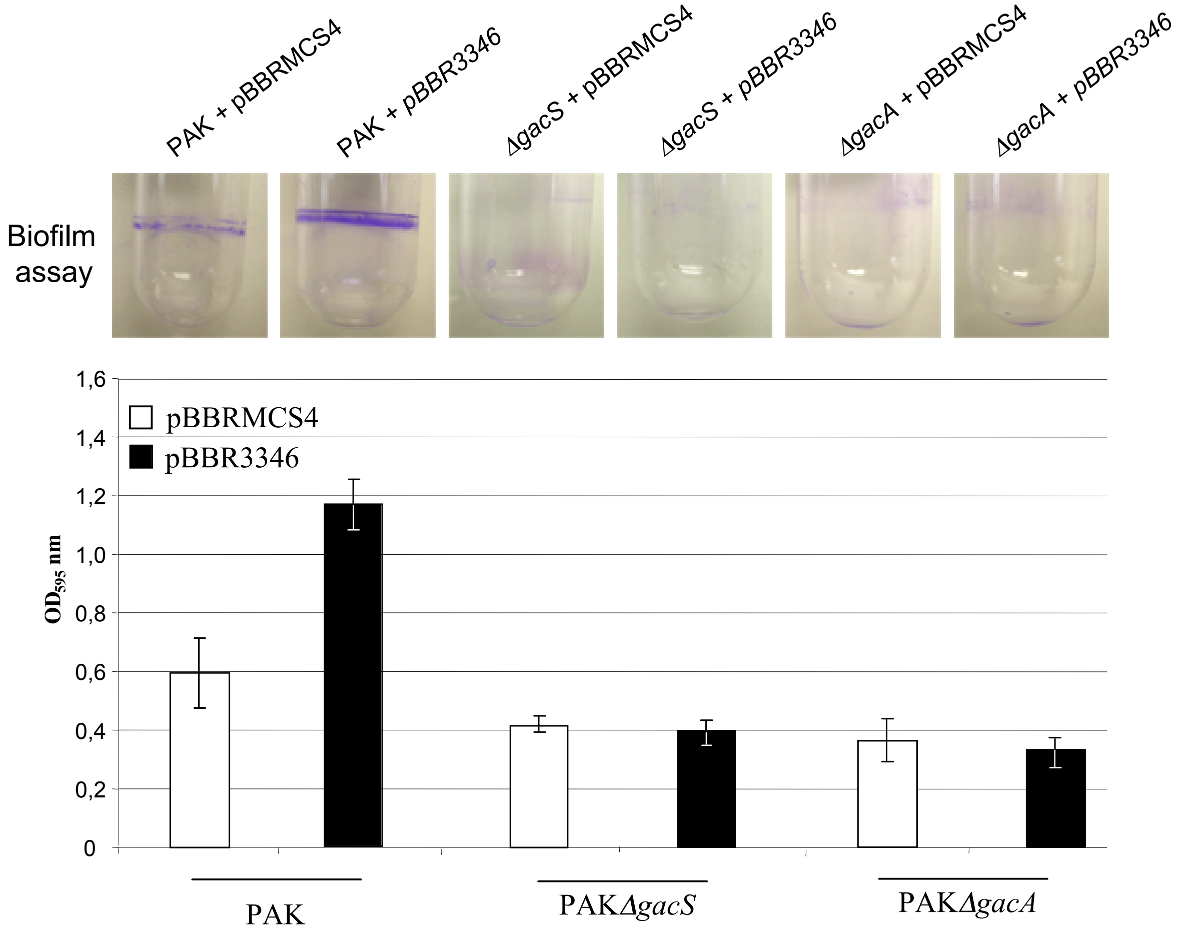

Fig. S4. Influence of *PA3346* overexpression in PAK, PAKΔ*gacA* or PAKΔ*gacS* strains on biofilm formation.

A. Glass tube assay showing biofilm formation. The name of the tested strains is indicated above each panel with *PA3346* expressed from the pBBR3346 plasmid whereas pBBRMCS4 is the cloning vector.

B. Quantification of the adherence ring formed in the glass tube. Each experiment was repeated three times. The error bars indicate standard deviations. The name of the strains used is indicated under each bar. Filled bars correspond to strains carrying pBBR3346 whereas open bars correspond to strains carrying pBBRMCS4. The pBBR3346 allowed overexpression of the *PA3346* gene cloned into the pBBRMCS4 vector.

Figure S5

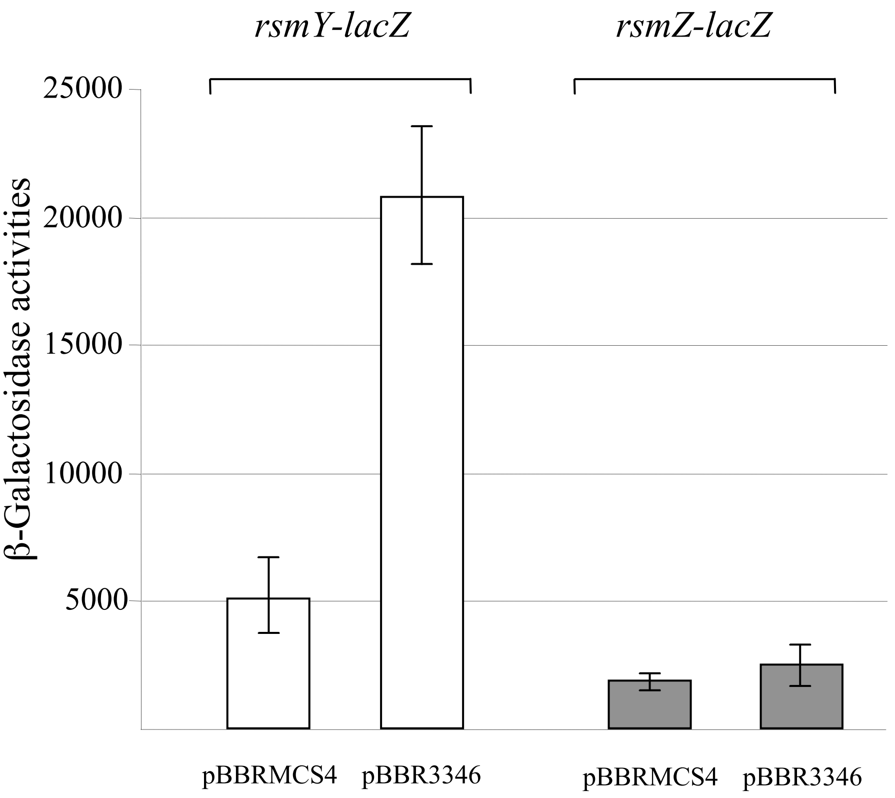

Fig. S5. Expression of the *rsmY* and *rsmZ* genes in *P. aeruginosa* strains overexpressing PA3346 or not. Activity of the *rsmY-lacZ* (open bars) and *rsmZ-lacZ* (filled bars) transcriptional fusion in PAK containing pBBRMCS4 or the derivative plasmid pBBR3346 overexpressing *PA3346*, was recorded after 4 hours of growth. β-galactosidase activities are expressed in Miller units. Values are averages of at least three independent experiments.

SUPPORTING INFORMATION

Table S1. Strains and plasmids used in this study.

Strains/Plasmids	Relevant characteristics[a]	Source/Reference
Strains		
P. aeruginosa		
PAK	Wild type	D. Bradley
PAKΔ*retS*	Deletion of *retS* in PAK	Goodman *et al.* (2004)
PAKΔ*hptB*	Deletion of *hptB* in PAK	This study
PAKΔ*PA3346*	Deletion of *PA3346* in PAK	This study
PAKΔ*PA3347*	Deletion of *PA3347* in PAK	This study
PAKΔ*gacA*	Deletion of *gacA* in PAK	This study
PAKΔ*gacS*	Deletion of *gacS* in PAK	This study
PAKΔ*rsmY*	Deletion of *rsmY* in PAK	This study
PAKΔ*rsmZ*	Deletion of *rsmZ* in PAK	This study
PAKΔ*pelB*	Deletion of *pelB* in PAK	Vasseur *et al.* (2005)
PAKΔ*hptB*Δ*pelB*	Deletion of *pelB* in PAKΔ*hptB*	This study
PAKΔ*hptB*Δ*gacA*	Deletion of *gacA* in PAKΔ*hptB*	This study
PAKΔ*hptB*Δ*gacS*	Deletion of *gacS* in PAKΔ*hptB*	This study
PAKΔ*hptB*Δ*rsmY*	Deletion of *rsmY* in PAKΔ*hptB*	This study
PAKΔ*hptB*Δ*rsmZ*	Deletion of *rsmZ* in PAKΔ*hptB*	This study
PAKΔ*hptB*Δ*rsmY*Δ*rsmZ*	Deletion of *rsmZ* in PAKΔ*hptB*Δ*rsmY*	This study
PAKΔ*htpB* Δ*PA3346*	Deletion of *PA3346* in PAKΔ*htpB*	This study
PAKΔ*htpB* Δ*PA3347*	Deletion of *PA3347* in PAKΔ*hptB*	This study
PAKΔ*retS*Δ*gacA*	Deletion of *gacA* in PAKΔ*retS*	This study
PAKΔ*retS*Δ*gacS*	Deletion of *gacS* in PAKΔ*retS*	This study
PAKΔ*retS*Δ*rsmY*	Deletion of *rsmY* in PAKΔ*retS*	This study
PAKΔ*retS*Δ*rsmZ*	Deletion of *rsmZ* in PAKΔ*retS*	This study
PAKΔ*retS*Δ*rsmY*Δ*rsmZ*	Deletion of *rsmZ* in PAKΔ*retS*Δ*rsmY*	This study
PAKΔ*retS* Δ*PA3346*	Deletion of *PA3346* in PAKΔ*retS*	This study
PAKΔ*retS* Δ*PA3347*	Deletion of *PA3347* in PAKΔ*retS*	This study
E. coli		
TG1	*supE* Δ(*lac-proAB*) *thi hsdR*Δ*5* (F' *traD36 rpoA*$^{+}$ *B*$^{+}$ *lacI*^{q} ZΔ*M15*)	Lab collection
TOP10F'	F'(*lacI*^{q} Tn*10*(Tet^{R})) *mrcA* Δ(*mrr-hsdRMS-mcrBC*) Φ80 *lacZ*Δ*M15* Δ*lacX74 recA1 araD139* Δ(*ara-leu*)*7697 galU galK rpsL*(Str^{R}) *endA1 nupG*	Invitrogen
DHM1	Reporter strain for two-hybrid system (F^{-}, *glnV44(AS), recA1, endA1, gyrA96 (Nal*^{r}*), thi1, hsdR17, spoT1, rfbD1, cya*)	Karimova *et al.* (2000)
CC118λpir	Propagation of pKNG101 plasmid and derivatives; Δ(*ara-leu*) *araD* Δ*lacX74 galE galK-phoA20 thi-1 rpsE rpoB argE*(Am) *recA1* Rf^{r} (λpir)	Lab collection
Plasmids		

pCR2.1	ColE1, f1 *ori*, Ap^R, Km^R	Invitrogen
pRK2013	Tra^+ Mob^+ Km^R	Lab collection
pMP220	Broad-host-range *lacZ* transcriptional fusion, Tc^r	Lab collection
p*pelA-lacZ*	pMP220 plasmid carrying a *pelA-lacZ* transcriptional fusion	Vasseur *et al*. (2005)
pSB307	pMP220 plasmid carrying a *exoS-lacZ* transcriptional fusion	Bleves *et al*. (2005)
pMP220*rsmZ-lacZ*	pMP220 plasmid carrying a *rsmZ-lacZ* transcriptional fusion	This study
pMP220*rsmY-lacZ*	pMP220 plasmid carrying a *rsmY-lacZ* transcriptional fusion	This study
pET-Dest42™	pET-Dest42™ , destination vector, LR recombination, V5 epitope and hexahistidine region (V5H6), ColE1, p*T7lac*, Ap^R, Cm^R, *ccdB*	Invitrogen
pET*vgrG1*	V5H6 tagged *vgrG1* cloned in pET-Dest42™	This study
pUCP18	Broad host range plasmid, Ap^R	Lab collection
pUCP*hptB*	pUCP18 carrying the *hptB* ORF	This study
pBBRMCS4	Broad host range plasmid, Ap^R	Kovach *et al*. (1995)
pBBR3346	pBBRMCS4 carrying the PA3346 ORF	This study
pBBR3347	pBBRMCS4 carrying the PA3347 ORF	This study
pKT25	Two-hybrid plasmid, cyaAT25 fusion, Km^R	Karimova *et al.* (2000)
pUT18C	Two-hybrid plasmid, cyaAT18 fusion, Ap^R	Karimova *et al.* (2000)
pKT25-*htpB*	Two-hybrid plasmid containing cyaAT25–*hptB* fusion	This study
pKT25-*PP2C*	Two-hybrid plasmid containing cyaAT25–PA3346 PP2C domain fusion	This study
pUT18C-*3346D*	Two-hybrid plasmid containing cyaAT18-PA3346 D2 receiver domain fusion	This study
pUT18C-*3347*	Two-hybrid plasmid containing cyaAT18–PA3347 fusion	This study
pKNG101	Suicide vector in *P. aeruginosa*; *SacB* St^r	Kaniga *et al*. (1991)
pKNGΔ*hptB*	Mutator plasmid for *hptB* deletion	This study
pKNGΔ*gacA*	Mutator plasmid for *gacA* deletion	This study
pKNGΔ*gacS*	Mutator plasmid for *gacS* deletion	This study
pKNGΔ*rsmY*	Mutator plasmid for *rsmY* deletion	This study
pKNGΔ*rsmZ*	Mutator plasmid for *rsmZ* deletion	This study
pKNGΔ*PA3346*	Mutator plasmid for *PA3346* deletion	This study
pKNGΔ*PA3347*	Mutator plasmid for *PA3347* deletion	This study
pKNGmamb3063	Mutator plasmid for *pelB* deletion	Vasseur *et al*. (2005)

[a] Ap^R ampicillin, Str^R streptomycin, Km^R kanamycin and Tc^R tetracycline

Table S2. Genes whose expression varies in *hptB* and *retS* background[a].

Gene expression varies both in PAKΔ*hptB* and PAKΔ*retS* mutant			Gene expression varies in PAKΔ*retS* mutant	
Genes[b]	Ratio in PAKΔ*hptB*	Ratio in PAKΔ*retS*	Genes[c]	Ratio in PAKΔ*retS*
pelB	3,19	4,46	**PA0084**	22,82
pelA	3,44	4,45	**PA0085**	9,12
exoS	-4,44	-53,7	PA0070	8,26
exoT	-6,14	-5,34	**PA0083**	7,66
exoY	-7,41	-16,01	**PA0086**	6,97
exsA	-5,1	-5,52	*arcC*	6,58
exsD	-5,84	-4,53	**PA0087**	5,88
PA1697 (PscN ATPase)	-2,29	-3,21	**PA0089**	5,63
PA2189	-2,32	-6,38	PA0126	5,05
PA3842 (Probable ExoS chaperone)	-2,23	-11,18	PA3729	4,78
PA3844	-2,60	-4,76	*pslA*	4,11
pcrD	-2,89	-2,92	PA3716	4,05
pcrH	-4,23	-14,98	PA0563	4,01
popB	-6,32	-41,24	PA5033	3,99
popD	-5,34	-10,5	PA1658	3,98
popN	-6,74	-5,39	PA3484	3,78
pscF	-3,18	-4,61	PA2537	3,73
pscJ	-5,19	-6,07	PA0095	3,70
pscL	-6,03	-4,65	*arcB*	3,58
			PA2204	3,39
			PA5136	3,25
			PA1068	3,10
			PA0277	3,08
			PA2581	3,04
			oprC	3,04
			**PA0078**	3,04
			norC	2,99
			PA4317	2,97
			PA4487	2,96
			PA0659	2,87
			PA3661	2,82
			PA0628	2,79
			PA0320	2,78
			PA3021	2,76
			PA0859	2,75
			PA0730	2,74
			PA1494	2,72
			sbp	2,71
			PA4491	2,67
			PA4495	2,61
			PA0045	2,57
			pslG	2,55
			PA1202	2,49
			PA0625	2,46
			metY	2,43
			PA4318	2,43
			PA3727	2,41

PA5530	2,39
oprH	2,36
PA0620	2,33
PA0201	2,32
PA1510	2,29
PA4321	2,21
PA3450	2,21
PA0434	2,19
PA1791	2,17
PA3022	2,14
PA1555	2,13
PA0141	2,12
PA4746	2,12
pykF	2,11
nosZ	2,07
PA0456	2,07
PA5178	2,05
nrdB	2,04
PA3931	2,02
PA2536	2,02
phoP	2,01
PA1132	2,00
PA0169	2,00
gbuA	-29,12
exsB	-5,49
hpd	-5,26
PA2776	-4,76
bkdA2	-4,56
PA3840	-4,47
bkdA1	-4,40
htpG	-4,22
dadA	-3,85
PA0779	-3,84
PA1418	-3,76
spuC	-3,76
PA1742	-3,60
gabD	-3,59
groEL	-3,59
putP	-3,51
pilV	-3,50
PA5312	-3,49
pscK	-3,46
PA1416	-3,28
pilE	-3,22
dnaK	-3,21
pilX	-3,21
PA1342	-3,16
pscD	-3,15
PA4506	-3,04
PA0296	-3,00
PA2040	-2,87
PA2016	-2,83
PA4505	-2,76
PA4504	-2,73

PA5436	-2,68
PA4503	-2,63
fimU	-2,62
PA0604	-2,60
prpC	-2,57
pilW	-2,57
hslU	-2,54
PA4918	-2,48
fahA	-2,47
gnyH	-2,38
spuB	-2,38
braC	-2,32
phhA	-2,28
PA4078	-2,26

[a]Genes down-regulated in the mutant versus the parental strain are displayed on green background, those up-regulated are on orange background.
[b]*exoS*, *exoT*, *exoY*, *exsA*, *exsD*, *exsD*, *PA1697*, *pcrD*, *pcrH*, *popB*, *popD, popN*, *pscF*, *pscJ, pscL* are type III secretion genes.
[c]Type VI secretion genes are written with thick characters.

Table S3. Oligonucleotides used in this study.

Name	Mutation/Cloning	Restriction site	Sequence 5'→ 3'[a]
BupA	*hptB* deletion	*Xba*I	GCTCTAGAAACCCTCAAGCATTTCAACCCGCTG
BupB	*hptB* deletion	*Eco*RI	TCCGAATTCTATCGCTGAGGTTTTCGTCCTTTCC
BloA	*hptB* deletion	*Eco*RI	ATAGAATTCGGACATTCGATCGCTCCCTGAAGTC
BloB	*hptB* deletion	*Spe*I	GGACTAGTGGTGCCAGGTAGAGCAGCTTGATCT
B5	*hptB* deletion		GAAGATCGGATATTCCTGTT
B6	*hptB* deletion		ACGATGCGGTAGAAGATGTT
Bup	*hptB* complementation		TTAAGCTTATGGGTAGGGCATCGGAAGT
Bdown	*hptB* complementation		AAGGATCCGACGAAAACCTCAGCGATAG
3346up	*PA3346* overexpression	*Eco*RI	CGGAATTCTGAACACACGTCTCTCCGTCG
3346down	*PA3346* overexpression	*Bam*HI	CGGGATCCGCTGGTAAAGGAAGGCCTTGG
3347up	*PA3347* overexpression	*Eco*RI	CGGAATTCACTGCTTACATGATTCAAGCC
3347down	*PA3347* overexpression	*Bam*HI	CGGGATCCTCAGCTGATCTTGAACAACTGC
UAgacA	*gacA* deletion	*Bam*HI	CGCGGATCCTAGTGCTGATCGGTGACGCC
LAgacA	*gacA* deletion	*Eco*RI	AAAGAATTCAATCACGCTGCACCTGCTCG
UBgacA	*gacA* deletion	*Eco*RI	AAAGAATTCTAGATGAGCGCCGTTTTCGA
LBgacA	*gacA* deletion	*Spe*I	GGACTAGTTCCTTCGAGGAACATCACCG
UAgacS	*gacS* deletion	*Bam*HI	CGGGATCCCGATCATGCTGGGTATGACCG
LAgacS	*gacS* deletion	*Eco*RI	CGGAATTCCACACGTCTCTCCGTCGAGCC
UBgacS	*gacS* deletion	*Eco*RI	CGGAATTCGAACTCTGACCATGCGCATCC
LBgacS	*gacS* deletion	*Spe*I	GGACTAGTCCGGTATTGATCAGCATCGC
UArsmY	*rsmY* deletion	*Bam*HI	CGCGGATCCAAACGGAACAGCTGGCTGGG
LArsmY	*rsmY* deletion	*Xho*I	CCGCTCGAGATTACGCATCTCTGCGAGGG
UBrsmY	*rsmY* deletion	*Xho*I	CCGCTCGAGTTATTGCCCGAGGAAAACCG
LBrsmY	*rsmY* deletion	*Spe*I	GGACTAGTTCGGGAATCGACATCGAGCG
UArsmZ	*rsmZ* deletion	*Bam*HI	CGCGGATCCTAGACGTCTCTCTTGGTCCG
LArsmZ	*rsmZ* deletion	*Xho*I	CCGCTCGAGCCTGCCGTTTTACTCGTCGC
UBrsmZ	*rsmZ* deletion	*Xho*I	CCGCTCGAGCCTGCCGTTTTACTCGTCGC
LBrsmZ	*rsmZ* deletion	*Spe*I	GGACTAGTGCACAAGCTGCTAGAATCGC
UAPA3346	*PA3346* deletion	*Bam*HI	GCGGATCCGCTTGGGACGGGCAGGTGGGAC
LAPA3346	*PA3346* deletion	*Xho*I	CCGCTCGAGGGGATTCATCTCAGCTGATCTTG
UBPA3346	*PA3346* deletion	*Xho*I	CCGCTCGAGTTCAAACAGGAACGTCAGCGC
LBPA3346	*PA3346* deletion	*Spe*I	GGACTAGTTGCAGGAAGGCCAGCATGCGCGG
UAPA3347	*PA3347* deletion	*Bam*HI	CGCGGATCCCCGGAGCGTCTGCAACGCTAT
LAPA3347	*PA3347* deletion	*Xho*I	CCGCTCGAGGGCCATGGAAGTCTCCTGGTA
UBPA3347	*PA3347* deletion	*Xho*I	CCGCTCGAGGTTGTTCAAGATCAGCTGAGA
LBPA33467	*PA3347* deletion	*Spe*I	GGACTAGTCGCCGAGCAGCACGTGCATGCC
PrsmY1	*rsmY* promoter	*Eco*RI	CGGAATTCAAGGCTCGCGATGATGAGG
PrsmY2	*rsmY* promoter	*Kpn*I	GGGGTACCTTTGGCGCTTCCTGCGCAATGTCC
PrsmZ1	*rsmZ* promoter	*Eco*RI	CGGAATTCCCTTAGACCCACTGAAGACC
PrsmZ2	*rsmZ* promoter	*Kpn*I	GGGGTACCATCCTTCGGGGTTGCGTGTTCC
UDHhptB	HptB for two hybrid	*Xba*I	GCTCTAGAGCGAATGTCCGCGCCGCATCTCGATGATCGTG

DDHhtpB	HtpB for two hybrid	*Kpn*I	GGGCCGGTACCTTGTCGCCGGAAAGGACGAAACCTCAGC
UDH3347	PA3347 for two hybrid	*Xba*I	GCTCTAGAGATGGCCATCACTGCGCTGCC
DDH3347	PA3347for two hybrid	*Kpn*I	GGGGTACCTCAGCTGATCTTGAACAACTGC
UDHPP2C	PP2C of PA3346 for two hybrid	*Xba*I	GCTCTAGACAACGTGCGCTACCTGCAATCG
DDHPP2C	PP2C of PA3346 for two hybrid	*Sac*I	CCGAGCTCCGACGAAAGCGCGATTATGCCTGAGG
UDHD3346	Receiver domain of PA3346 for two hybrid	*Xba*I	GCTCTAGATGAGATGAATCCCCCGGCGG
DDHD3346	Receiver domain of PA3346 for two hybrid	*Eco*RI	AACGGAATTCTCACGGCGAGAAATACTGGTTTCG
L1R1	Microarray construction		ACAAGTTTGTACAAAAAAGCAGGCT
L2R2	Microarray Construction		ACCACTTTGTACAAGAAAGCTGGGT

[a]Restriction site sequence is underlined.

SUPPORTING INFORMATION

References

Kovach, M.E., Elzer, P.H., Hill, D.S., Robertson, G.T., Farris, M.A., Roop, R.M., and Peterson, K.M. (1995). Four new derivatives of the broad-host-range cloning vector pBBR1MCS, carrying different antibiotic-resistance cassettes. *Gene* **166**, 175–176.
